# Excited-State Rotational Dynamics of Amine-Functionalized Terephthalic Acid Derivatives as Linker Models for Metal–Organic Frameworks

**DOI:** 10.1021/acs.jpca.4c03827

**Published:** 2025-01-15

**Authors:** George Healing, Maksim Zakharzhevskii, Issatay Nadinov, Luis Gutiérrez-Arzaluz, Shorooq A. Alomar, Jorge Gascon, Omar F. Mohammed

**Affiliations:** † Advanced Membranes and Porous Materials Center, Division of Physical Science and Engineering, King Abdullah University of Science and Technology, Thuwal 23955-6900, Kingdom of Saudi Arabia; ‡ KAUST Catalysis Center, Division of Physical Sciences and Engineering, King Abdullah University of Science and Technology, Thuwal 23955-6900, Kingdom of Saudi Arabia

## Abstract

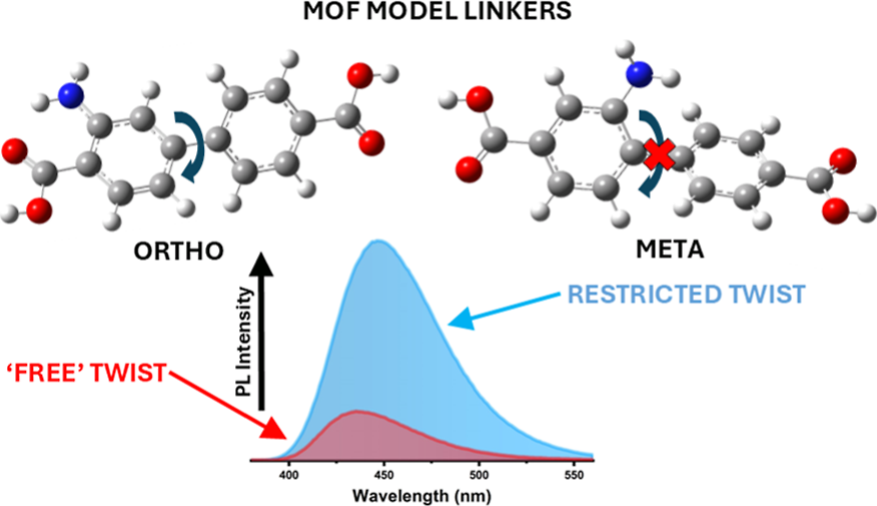

Understanding how structural modifications affect the photophysics of organic linkers is crucial for their integration into metal–organic frameworks (MOFs) for light-driven applications. This study explores the impact of varying the amine functional group position on two terephthalic acid derivatives—linker **1** and linker **2**—by investigating their photophysics through a combination of steady-state and ultrafast laser spectroscopy and time-dependent density functional theory (TD-DFT) calculations. With tetrahydrofuran as the solvent, time-correlated single-photon counting revealed a 2-fold increase in the S_1_ excited-state lifetime of the molecule with the amine group at the meta position compared with that of the molecule with the amine group at the ortho position. This phenomenon can be attributed to restricted intramolecular twisting for the molecule with the amine group in the meta position. In this regime, an interplay of high-energy steric and conjugation barriers was revealed for the molecule with the amine group at the meta position by TD-DFT calculations. Moreover, femtosecond/nanosecond transient absorption spectroscopy revealed a reversible excited-state conformational change for the ortho isomer via intramolecular rotation that occurred within ∼110 ps, unlocking a triplet state manifold. This study underscores the importance of modifying organic emitters, either as free linkers or within MOFs, to increase their performance in sensing and light-emitting applications.

## 1 Introduction

Successful applications of metal–organic frameworks (MOFs) include the tunability and careful design of organic building blocks that constitute their chemical structures. The use of careful chemical manipulation of the MOF building blocks to influence their properties was reported for the first time in the early 1990s and has since evolved into a thriving research area.^1,2^ A deep understanding of the fundamental chemistry that governs organic and inorganic components allows researchers to make informed decisions to achieve the desired outcomes for their specific applications. For example, understanding of the structure–property relationships of luminescent organic free linkers provide opportunities for enhancing their absorption and emissive characteristics. Understanding of the photophysical properties of the free linkers provides a valuable foundation for predicting the response of the MOF to light, energy transfer, or charge separation. This knowledge can help tailor the properties of the target MOF for specific applications, such as photocatalysis or light-emitting diodes (LEDs). Thus, an in-depth examination of the photophysics of free linkers prior to their incorporation into MOFs enhances the understanding of the excited-state dynamics and distinct impact of individual components, which are crucial factors in photoinduced processes and applications.^3^

Terephthalic acid is a common organic linker in MOFs, largely due to its size and terminally situated carboxylic acid functional groups which allow facile coordination with inorganic/metal nodes.^4^ Its aromatic central ring and length provide adequate pore space, rigidity, and additional structural stability between the coordination sites.^5^ Thus, the use of terephthalic acid as an archetype for molecular customization has afforded a rich variety of derivative linkers for numerous MOF applications, from CO_2_ absorption to wastewater treatment.^6,7^ For example, a derivative known as 2-aminoterephthalic acid has gained popularity as a photosensitizer in MOFs employed for visible-light photocatalysis.^8−11^ Its polar amino group extends into pore spaces without bonding to metal nodes, enabling it to participate in numerous excited-state electron-transfer processes between substrates, linkers, and metal clusters. This characteristic makes it a favored choice in photocatalytic applications.^12,13^

Generally, photocatalytic MOFs should ideally absorb in the visible range, because such absorption is essential for efficient solar energy utilization. In this case, the absorption of 2-aminoterephthalic acid can shift to the visible range, and 2-aminoterephthalic acid can subsequently undergo linker-to-metal charge transfer (LMCT) via its aromatic ring or proximal amine.^14,15^ Then, the reduced form of the metal can reduce a targeted substrate. Therefore, optimization of certain photophysical properties of these types of organic linkers, such as the excited-state lifetime or LMCT efficiency, introduces possible routes for improving the performance of these photocatalytic MOF systems.

**Figure 1:**
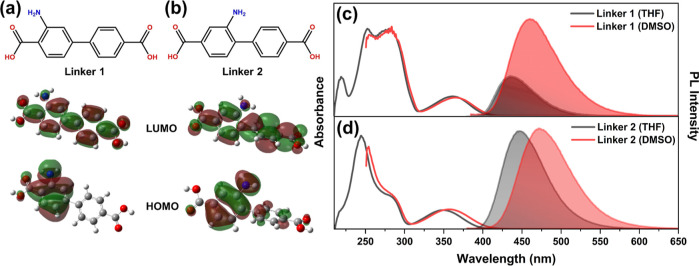
Linkers **1** (a) and **2** (b) with their frontier molecular orbitals. Absorption (solid lines) and emission (filled curves) spectra of (c) **1** and (d) **2** in THF and DMSO solutions.

Among various organic linkers are two aminoterephthalic acid derivatives, namely 3-amino-[1,1′-biphenyl]-4,4′-dicarboxylic acid (linker **1**) and 2-amino-[1,1′-biphenyl]-4,4′-dicarboxylic acid (linker **2**). These derivatives feature an extra aromatic ring and differ structurally due to the different positions of the amino function group (namely ortho and meta, see Figure 1a,b). These two linkers have been used in many applications, such as molecular adsorption, photocatalytic reduction of CO_2_ and H_2_O, ratiometric sensing, and hydrolysis of toxic chemicals.^16−21^ In these linkers, the double-ring system not only serves to increase the pore size but can also redshift the spectral absorption further into the visible range. To the best of our knowledge, a comprehensive photophysical study of these linkers has not been conducted to date. Specifically, in the free form (i.e., not incorporated into a MOF), the position of the amine group can strongly influence the lifetime of the linker itself beyond its LMCT capability. This hypothesis particularly considers the steric hindrance introduced when the amine group is positioned in the meta rather than the ortho position. By limiting the extent of intramolecular rotation in the excited state, nonradiative relaxation to the ground state can be reduced, thereby prolonging the lifetime of the excited state.^22^ These intramolecular rotational effects can also occur when the linker is integrated into a MOF but only to a certain extent, further supporting the importance of studying the linker prior to its integration.^23^ Another method of extending the excited-state lifetime is based on the population of accessible triplet states, which is well-known to exhibit a slower phosphorescence phenomenon because of their forbidden transition to the ground state.^24,25^ Understanding the relevant structure–function relationships provides another avenue for the improvement in the efficiency for both preintegration and postintegration into a MOF.

Herein, we explored the rotational dynamics of free linkers **1** and **2** (shown in Figure 1a–b) using a combination of steady-state and femtosecond/nanosecond transient absorption (fs/ns-TA) spectroscopy, time-correlated single-photon counting (TCSPC), and time-dependent density functional theory (TD-DFT). Steady-state photoluminescence (PL) measurements revealed variations among the linkers not only in the same solvent but also in different solvents, specifically, dimethyl sulfoxide (DMSO) and tetrahydrofuran (THF). The increased PL observed for linker **2** in DMSO suggested that viscosity may play a crucial role in restricting intramolecular rotation. TCSPC measurements supported this hypothesis by revealing longer lifetimes for linker **2**. The rotational energies calculated using TD-DFT further confirmed these results, underscoring the impact of steric hindrance and electronic conjugation in limiting rotation in the excited state and subsequently suppressing nonradiative channels. Examination of the accessible triplet states via fs/ns-TA spectroscopy provided additional insights into the photophysics of these organic linkers. Thus, this study considerably advances our understanding of the structure–function relationships in MOF-based photocatalytic systems and offers valuable insights for enhancing their performance.

## 2 Results and Discussion

### 2.1 Steady-State and Excited-State Lifetime Measurements

First, the steady-state absorption spectra (Figure 1c,d) at the same concentration of 1 mM in THF and DMSO were measured. The key solvent properties of THF and DMSO are listed in Table 1. The absorption spectra for **1** and **2** show a similar *n*–π* transition at ∼278 nm in addition to a π–π* transition at 350 nm.^26^ A secondary π–π* transition is also observed for both samples at 255 and 243 nm for **1** and **2**, respectively; however, it is more intense for **1**. A comparison of the experimental and calculated spectra is shown in Figure S14. The emission spectra presented in Figure 1c,d were obtained under similar conditions at a concentration of 1 mM. Linker **1** exhibited a broad Gaussian line shape in the PL spectra for both solvents, with a redshift from 435 to 460 nm when it was changed to a more polar solvent (THF to DMSO). Similarly, linker **2** exhibits a broad emission line shape that redshifts from 446 to 473 nm as the solvent polarity increases. Combined with the broad Gaussian line shape of the PL, the redshift in emission with increasing solvent polarity strongly suggests the presence of excited-state charge transfer (CT) for both linkers.^27^

**Table 1:** Physical Properties of the Solvents^28^

solvent	dielectric constant, 25 °C	polarity index	viscosity η × 10^–3^, 20 °C (kg m^–1^ s^–1^)	donor number	acceptor number
dimethyl sulfoxide	46.7	7.2	2.0	29.8	19.3
tetrahydrofuran	7.6	4.0	0.6	20.0	8.0

Time-dependent DFT calculations supported the CT character of the transition by revealing the dominant molecular orbitals involved in the ground-state to excited-state transition (Figure 1a,b). The highest occupied molecular orbital (HOMO) for **1** and **2** was localized on the left ring fragment containing the amine functional group, although with a slight π orbital contribution from the adjacent aromatic ring in linker **2**. Excitation to the excited state occurred through a π–π* transition into the lowest unoccupied molecular orbital (LUMO) that was distributed throughout the entire molecule in both Linkers **1** and **2**. Previous computational studies demonstrated comparable findings for aminoterephthalic acid, specifically indicating a decrease in the contribution of the amine functional group to the LUMO state.^29^

**Figure 2:**
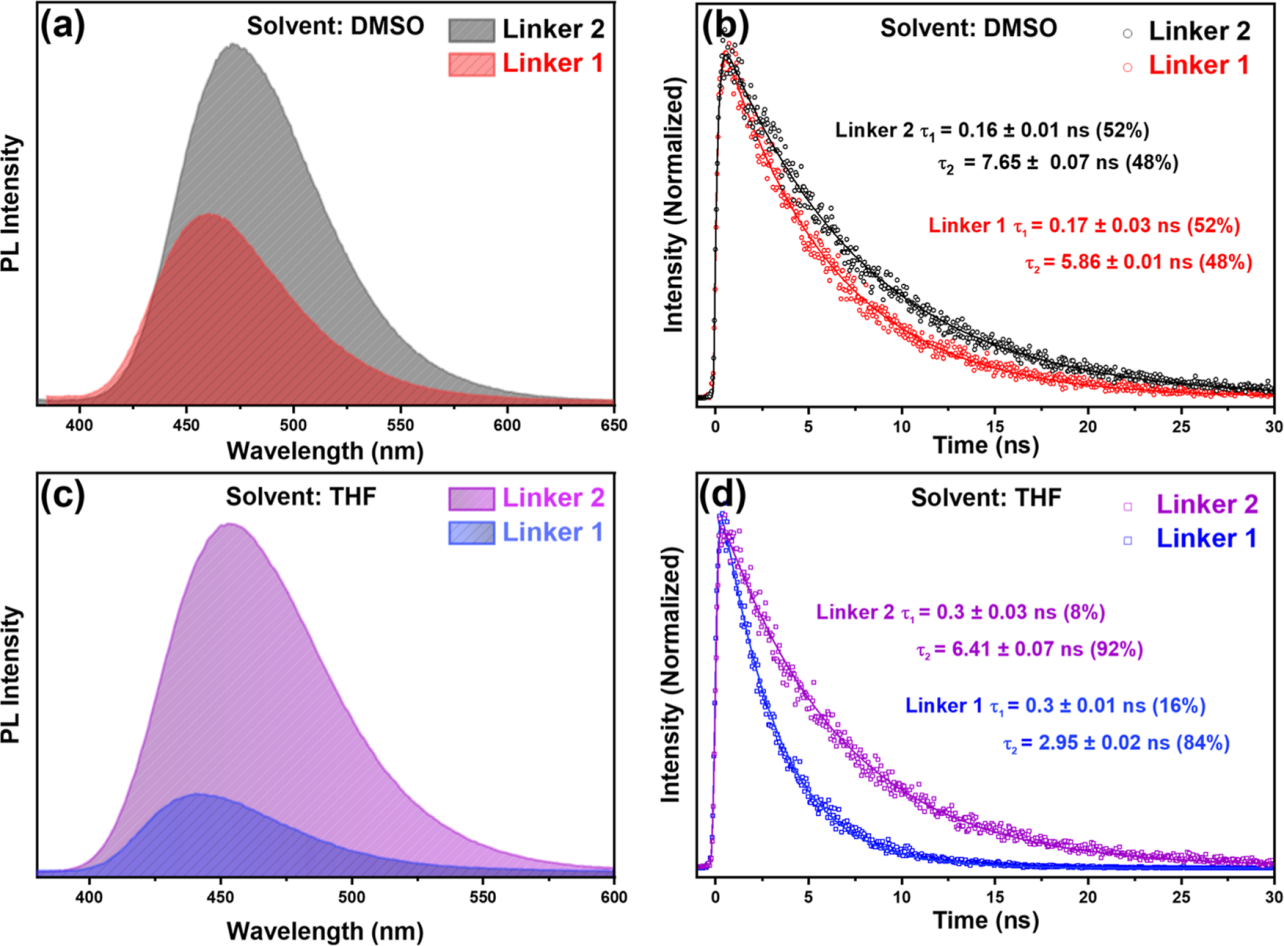
Steady-state PL for linkers **1** and **2** in (a) DMSO and (c) THF. TCSPC decays for **1** and **2** in (b) DMSO and (d) THF with the time constants for the decay fits shown in the inset (λ_exc_ = 350 nm).

In Figure 1, the spectra compare the effects of two solvents with different polarities for a single linker. In contrast, Figure 2 compares the PL and lifetime of each linker in the same solvent, enabling a direct assessment of the impact of the amino group position. Notably, the comparison reveals a considerably higher PL intensity for linker **2** than for linker **1**. In THF, the PL intensity of linker **2** was 4.5 times greater than that of linker **1**, whereas this difference was 1.9 times higher in DMSO. A shift in the emission peak within the same solvent was also apparent. In DMSO, the emission peak for linker **2** red-shifted to 473 nm relative to the 460 nm peak for linker **1**, indicating a correlation between the emission wavelength and position of the amino functional group. A similar redshift was observed in THF, with a shift from 441 to 455 nm from linker **1** to linker **2**, suggesting potential steric and/or electronic-structure differences between the two linkers as the factors contributing to these spectral differences.

The steady-state absorption measurements show that at least according to the Beer–Lambert law, both Linkers **1** and **2** have similar molar absorptivities at 350 nm in both solvents (∼2160 M^–1^ cm^–1^).^30^ Given similar photon absorption, variations in the emission intensity indicate the presence of variations in the excited-state emission dynamics. To delve deeper into this phenomenon, we used time-resolved PL measurements employing TCSPC. The time-resolved fluorescence decay profiles for Linkers **1** and **2** in DMSO and THF are presented in Figure 2b,d, respectively, along with their respective average weighted time constants.

All fluorescence decay profiles for Linkers **1** and **2** in both solvents could be effectively modeled using a double exponential decay function. Figure 2b shows the fluorescence decay of linkers **1** and **2** in DMSO, demonstrating a longer average lifetime for linker **2** (3.91 ± 0.04 ns) than for linker **1** (3.02 ± 0.02 ns). The contrast in the lifetime between linkers **1** and **2** in THF (Figure 2d) was more pronounced, with values of 1.58 ± 0.05 and 3.29 ± 0.07 ns, respectively. The time-resolved photoluminescence (PL) obtained via time-correlated single-photon counting (TCSPC) were normalized to facilitate comparisons across conditions. While the time resolution of TCSPC limits the detection of subpicosecond dynamics, femtosecond transient absorption (fs-TA) measurements were conducted to capture ultrafast processes. It is important to note that relative differences in PL quenching may not perfectly align with their corresponding changes in lifetime. Although steady-state PL intensity and time-resolved PL (TRPL) lifetime are proportional, the steady-state PL intensity is both time- and spectrally integrated, meaning it is influenced by fluorescence lifetime as well as spectral width and shape. Consequently, single-wavelength TRPL measurements, while correlated with steady-state PL, may exhibit deviations due to spectral broadening or shifts that affect the integrated intensity.

Given the same conditions and similar absorption coefficients, if one species exhibits a greater PL than the other, we can infer that the difference is due to either radiative or nonradiative losses. The lifetime (_s_) of an excited state S_1_ can be given by
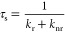
1where *k*_r_ and *k*_nr_ are the radiative and nonradiative rate constants, respectively. As TCSPC lifetime measurements show the lifetime of the S_1_ states, we can therefore, in conjunction with the PL intensity, qualitatively infer the relative contribution of each rate constant. The increase in the lifetime from linker **1** to linker **2** can be attributed to an increase in the radiative and/or nonradiative rates in the denominator, as indicated by eq 1. *k*_r_ cannot be solely responsible for this increase in the lifetime because we observed a remarkable increase in the PL intensity for **2** compared with that for **1**. Additionally, if both rates were to increase, we would expect a much greater disparity between the lifetimes of linkers **2** and linker **1**; combined with the higher PL intensity for linker **2**, this suggests that nonradiative decay pathways (*k*_nr_) play a more significant role in linker **1**. To summarize, a longer lifetime typically correlates with a lower *k*_nr_, as nonradiative processes shorten the excited-state lifetime. Since linker **2** has a longer lifetime and a higher PL intensity, its *k*_nr_ is reduced compared with that of linker **1**. Conversely, the shorter lifetime and lower PL intensity of linker **1** suggest that nonradiative processes are more dominant in linker **1**.

Comparison of the spectra of linker **1** in different solvents shows that for linker **1**, the steady-state emission is even lower in THF than in DMSO, which is consistent with the trend observed in lifetimes where a much shorter lifetime is observed. This observation implies that, compared with linker **2**, linker **1** is losing more of its excited-state energy to nonradiative pathways. In summary, when comparing linkers **1** and **2** for the same solvent, the difference between their lifetimes may be attributed to the steric hindrance caused by the amino group. Specifically, the increased rigidity of a molecule can result in a decrease in its nonradiative rate.^31^ For the same linker in different solvents, the differences between the lifetimes are likely due to the viscosity effects of the solvents that lead to restricted intramolecular rotation. The Förster-Hoffmann equation was used to describe the fluorescence lifetime dependence on solvent viscosity, highlighting differences in sensitivity between linker **1** (*n* = 0.54) and linker **2** (*n* = 0.12).^32^ These values indicate a moderate viscosity dependence for linker **1** and a weaker dependence for linker **2**. This analysis, along with the log plot, can be found in Supporting Information S1.

Furthermore, the potential impact of any aggregation effects on the excited-state dynamics was explored. The formation of aggregates at high concentrations may lead to phenomena such as aggregation-induced enhanced emission, aggregation fluorescence quenching, or excimer formation.^33^ However, TCSPC lifetime measurements from high to low concentrations (i.e., 1–0.02 mM) (Figure S2) indicated no significant differences in the lifetime between the two linkers at different concentrations.

The rotation of the central C–C bond axis can play an important role in the excited-state dynamics of organic linkers.^23^ However, restriction of bond rotation is influenced not only by steric hindrance but also by electronic conjugation, such as the presence of π bonds. Both linkers are anticipated to demonstrate delocalized character across the central C–C bond in both the ground and excited states. These factors, along with the impact of steric hindrance caused by the amino groups, contribute to rotation restriction. To delve deeper into the relative contributions of each of these phenomena, further investigation through TD-DFT calculations is warranted.

### 2.2 Computational Study

**Figure 3:**
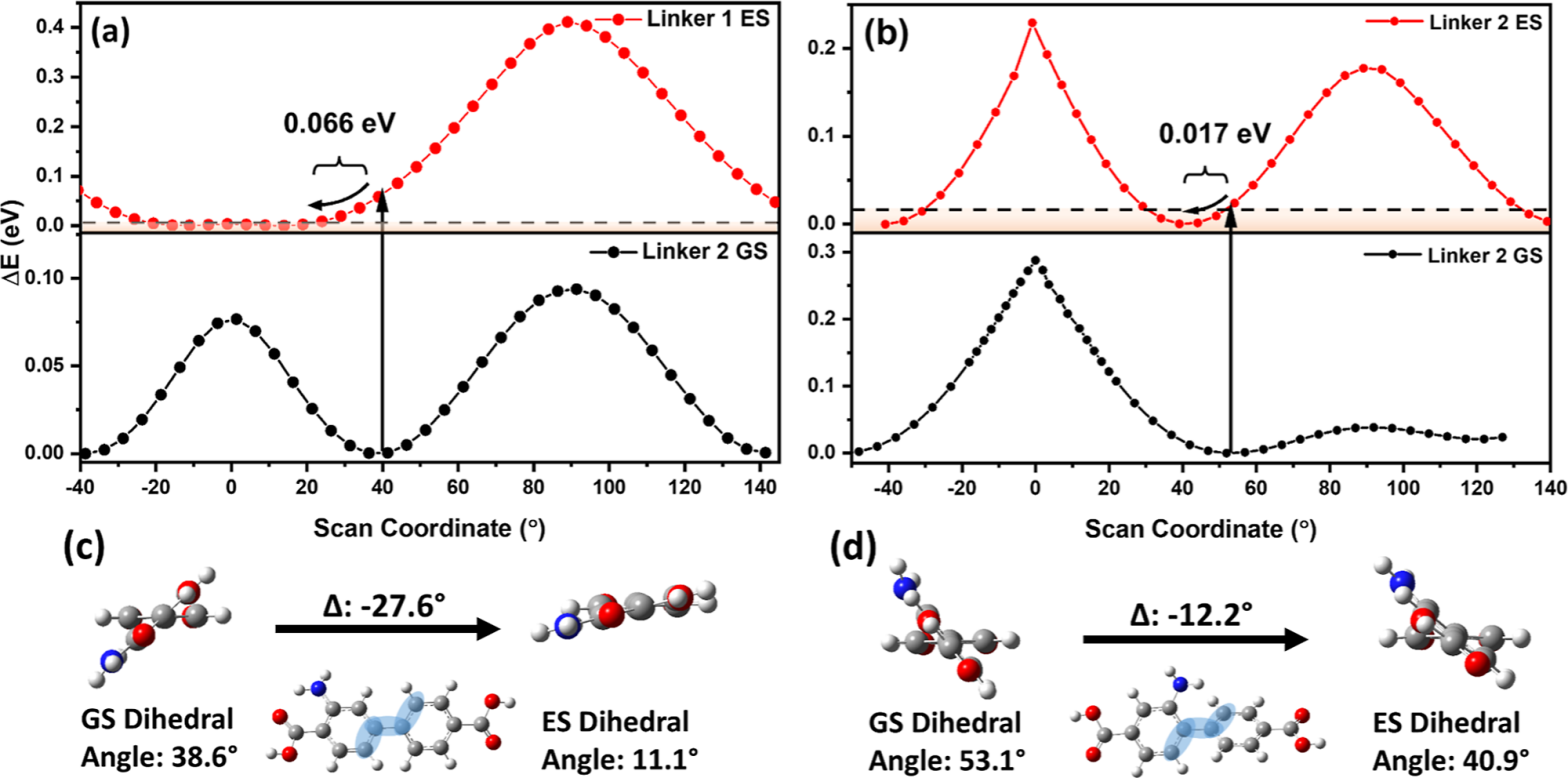
Energy curves for partially optimized linker structures following incremental changes in the dihedral angle between the two phenyl rings for (a) linker **1** and (b) linker **2** in the ground and excited states. The dashed horizontal line indicates the average thermal energy (*k*_B_*T*) at room temperature and pressure. (c,d) GS- and ES-optimized geometries of **1** and **2**, respectively, with changes in their intraring dihedral angles.

In addition to the influence of the steric hindrance of the amino group on the degree of twisting motion, orbital contributions across the C–C bond linking two aromatic ring fragments may also play a significant role. The question that remains at this point is to what extent electronic conjugation may also influence restricted rotation. To help further elucidate the detailed mechanistic origins of the photophysical differences between **1** and **2**, TD-DFT was conducted on the two linkers. First, the geometries of both **1** and **2** were optimized as singular units in the ground state with THF as an implicit solvent (Figure 3).

Analysis of the optimized structures revealed that the dihedral angles between adjacent rings were 38.6 and 53.1° for Linkers **1** and **2**, respectively. These angles reflect a delicate balance between the increase in energy from steric effects and the reduction in energy from conjugation effects. The larger dihedral angle in linker **2** is attributed to the greater steric hindrance caused by the meta-amino group, in contrast to the minimal hindrance from a hydrogen atom in the same position on the aromatic ring in linker **1**. The sharp change in the potential energy curve at approximately 0° is due to the large increase and subsequent release of potential energy due to the proximity of an amine hydrogen with an aromatic hydrogen on the adjacent ring.

Although both molecules would ideally prefer a planar arrangement to enhance conjugation between the rings, such an arrangement is prevented by the presence of steric hindrance at the intersection. To further understand the interplay between these opposing effects, a scan calculation was conducted to determine the energies and contributions of each effect to the optimization process. A series of stepwise partial optimizations were conducted at 5° increments, in the positive and negative directions from the optimized ground-state dihedral angle for each linker (Figure 3a,b), obtaining a potential energy surface for rotation around the central axis in both excited and ground states. Notably, two peaks for each plot corresponded to the energy barriers of increased steric hindrance and breaking conjugation. The steric barrier was located at ∼0° and represented the maximum increase in energy when the rings were completely coplanar and when hydrogen atoms (amino groups in the case of linker **2**) came into close proximity. At ∼ 90°, the energy barrier was associated with breaking of the electronic conjugation between the two phenyl rings. To simulate the rotational dynamics in the excited state, the Franck–Condon principle was utilized. This principle asserts that given the instantaneous nature of electronic transitions, the subsequent evolution of nuclear motion occurs on a longer time scale than the initial excitation.

The energy difference between the current state and the optimized excited state was related to the release of excess energy associated with the initial twist around the inter-ring bond. A twisting energy barrier of 0.066 eV was obtained for linker **1** (Figure 3a), and 0.017 eV was obtained for linker **2** (Figure 3b), indicating greater energy release for linker **1** during energy minimization in the excited state. The twisting energy barriers in the excited state are highlighted by the red curves in Figure 3a,b. The steric barrier almost completely disappeared for linker **1**, enabling free rotation by ∼43° at room temperature, as highlighted by all the points below the dashed line at the *k*_B_*T* value. For linker **2**, free rotation was possible for only ∼25°, as the rotation was more highly constrained between the two adjacent energy barriers. Importantly, the inclusion of thermal energy *k*_B_*T* in the excited-state potential energy surface, but not in the ground state, was intended to illustrate two key points: first, the extent to which either linker can rotate at room temperature relative to its energy barriers, and second, that the molecule cannot overcome these barriers at room temperature energies. While electronically excited molecules typically possess kinetic energies greater than thermal energy, incorporating *k*_B_*T* into the excited-state PES helps highlight the available rotational freedom and the constraints imposed by thermal energy.

Based on these computational results, the conjugation between the two ring systems in both linkers was apparently a key factor in restricting rotation. Breaking the conjugation in linker **1** resulted in a considerably higher energy barrier, as seen in the elevated potential barrier in the excited state, compared with that in linker **2**. This enhanced conjugation between the rings motivated a deeper exploration of the quinoidal excited state and the impact of mesomeric structures, such as those due to charge delocalization, on this state. Laplacian bond order analysis of the inter-ring C–C bonds revealed an increase in bond order from 1.09 to 1.23 from the ground state to the excited state in linker **1** and an increase from 1.06 to 1.28 in linker **2**. This indicates an increase in quinoidal character in the excited state, a phenomenon that is quite common in conjugated ring systems.^34,35^ This increase in quinoidal character explains the increase in the height of the conjugation barrier for linker **2** in the excited state. However, it does not explain the much larger increase for linker **1**. Using modified Mulliken population analysis, we quantified the difference in the charges between the two rings for the transition from the ground state to the excited state.^36^ For linker **1**, the right (nonamine-containing) fragment gained 3.67 au of negative charge, whereas the left (amine-containing) fragment for linker **2** gained a charge of 2.27 au, i.e., linker **2** was less able than linker **1** to delocalize the charge in the excited state. Notably, the CT state was mostly distributed among the entire molecule for both Linkers **1** and **2**. Previous work on meta-amino benzoic acid has shown that this meta configuration can have a preference for strong CT between the amino and carboxylic acid functional groups.^37^ This finding indicates that for **2**, the CT had a more localized contribution (within the same ring) compared to that for **1**. Electron density difference plots (Figure S3) confirm this observation, highlighting the increased electron density on both oxygen atoms in the neighboring carboxylic acid group for linker **2** in contrast to a minimal contribution for linker **1**. The increased electron density from the lone pairs of the amino group in the ortho position likely plays a significant role in the greater enhancement of conjugation between the two rings upon entering the excited state.

Hence, while the conjugation barrier in the excited state was considerably greater for linker **1**, the steric hindrance was decreased, enabling unrestricted rotation between the conjugation barriers. In the case of linker **2**, while there was a similar increase in conjugation upon entering the excited state, the presence of the amino group in the meta position maintained the steric obstacle. Consequently, rotation was more constrained between the two barriers in linker **2**. Franck–Condon analysis further demonstrated greater energy release from rotation in linker **1** upon transition to the excited state, as the vertical transition into an energetically unstable state initially induced the rings to rotate into their optimized geometries. These computational findings confirm the hypothesized impact of steric hindrance.

### 2.3 fs/ns-Transient Absorption Spectroscopy

**Figure 4:**
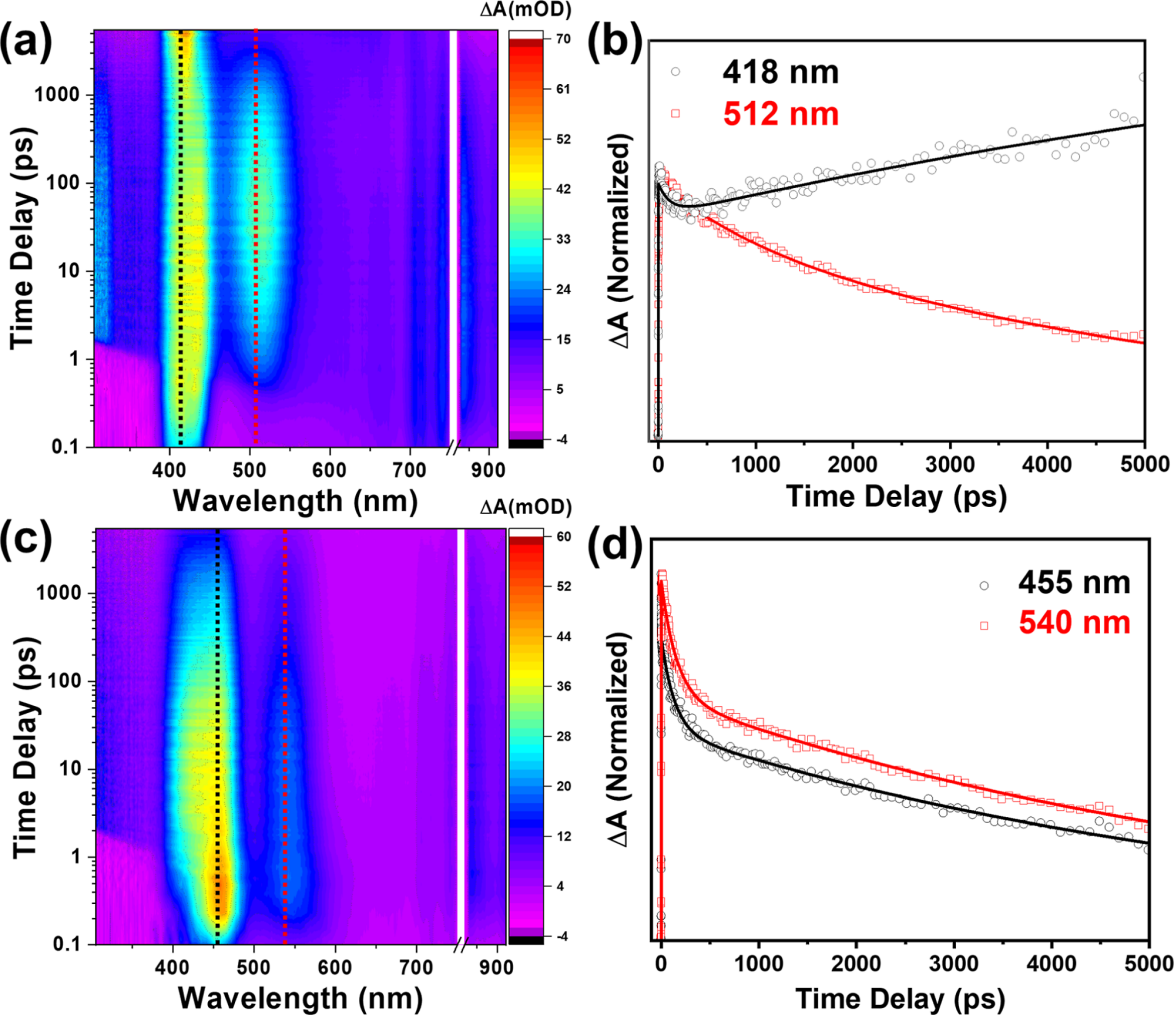
2D color contour plots of the fs-TA spectra for the linkers in THF. Linker **1** (a) with (b) a kinetic plot comparing peaks at 418 and 512 nm and (c) linker **2** with (d) a kinetic plot comparing peaks at 455 and 540 nm. (λ_exc_ = 350 nm).

To observe the rotational dynamics of the linkers in the excited state, the evolution of the electronic excited state must be probed. For this purpose, TA spectroscopy (TAS) was used because any changes in the linker’s excited-state geometric structure are reflected in its electronic structure. Linker **1** showed four main absorption features in the TA spectrum (Figure 4a). The peak at ∼425 nm consists of two intense overlapping excited state absorption (ESA) peaks at 418 and 438 nm. The third overlapping peak is a singular ESA peak at 512 nm, and another broad ESA signal that extends from ∼700 to 870 nm is also observed. All of the peaks decayed with a typical biexponential function except for the primary peak at 418 nm, which was fitted with a triexponential function and exhibited an increase at ∼110 ps (see the inset of Figure 4a). Additionally, a very weak and broad signature at 650 nm was observed that exhibited the same triexponential fit and an increase after ∼110 ps, similar to the absorption at 418 nm (see Figure S4). Based on the steady-state spectra presented in Figure 1, the features at approximately 460–470 nm are candidates for a stimulated emission (SE) signal. Our global analysis and decay-associated difference (DAD) spectra (Figure S13) indicate the presence of a broad SE signal underlying the excited-state absorption (ESA) signals; however, it decays and transitions to ESA after 1.07 ps. Therefore, we believe that these bands represent distinct spectral features rather than a single band influenced by SE.

The TA spectrum for linker **2** in Figure 4b shows four main peaks: two intense overlapping ESA peaks at 428 and 455 nm, a single ESA peak at 540 nm, and a broad ESA signal between 710 and 860 nm. For all of the peaks, the kinetics can be fitted with a triexponential function and the function parameters are listed in Tables S1–S6. Comparing Linkers **1** and **2**, similar spectral shapes are observed for the two species: a strong double-peak feature between 418 and 455 nm, a separate broader peak at 512–540 nm, and a very broad feature at >700 nm. This spectral pattern reflected the similarity of the electronic structures of Linkers **1** and **2**. The origin of the peaks for linker **2** can be explained by the CT nature of its excited state. Coupled with the larger dihedral angle of linker **2**, the fast rise times (1.9 and 6.0 ps, respectively) of the 455 and 865 nm peaks suggest that these two states are associated with a twisted intramolecular CT (TICT) state.^38^ The slower rise times of the 428 and 540 nm peaks therefore suggest that these are relaxed S_1_ states (RS_1_) that are occupied after energy redistribution rather than being directly excited by the pump.

The major difference between the two linkers lies in the sudden rise in the decay profile for the 418 nm peak and the apparent formation of a new peak at 650 nm for linker **1**. This change in dynamics can be explained in several ways. One possible explanation is the formation of side products through the laser-induced breakdown of the linker. However, this was ruled out, as the intensity of the peaks did not change after multiple scans in the TA experiment. The second possibility is that a change in the conformation of the linker causes a different electronic structure. Considering the increased freedom of linker **1** to rotate around its central axis, energy may be redistributed following the change in the excited-state conformation. This redistribution would then allow repopulation of the RS_1_ state at 418 nm as the molecule became more planar. This hypothesis is supported by the fact that the decay profile for the two peaks began to rise again at approximately the same time with similar time constants. Apparently, the excited state of both linkers extended beyond the time window of the TA experiment, namely, the two rising components for linker **1**.

The region at approximately 418 nm is complex due to the potential spectral overlap between the excited-state absorption (ESA) and stimulated emission (SE) features, as indicated in Figure 1c,d. To address this issue, we performed a global analysis of the TAS kinetics across the entire spectral range. The associated DAD spectra can be found in Figure S13. For linker **1** (Figure S13a), the 418 nm peak initially decays within 1.06 ps but then significantly increases beyond the range of the fs-TA measurements. Additionally, a strong stimulated emission (SE) that decays over the same time scale is evident between 440 and 560 nm. The excited-state absorption (ESA) peak visible in the normal TA spectrum also appears at 440 and 520 nm at 2.8 ns. Furthermore, it is clear that at 2.8 ns, the influence of the SE disappears between 440 and 560 nm. For linker **2** (Figure S13b), a strong SE signal at 420 nm decays rapidly within 1.3 ps, accompanied by a potential SE signal at 525 nm, which is convoluted with the strong ESA peaks. These signals likely represent the same SE feature, with a prominent ESA peak at 460 nm in the middle. Consequently, care must be taken when interpreting dynamics within the first 1.3 ps.

Repeating the same measurements in DMSO shows that for linker **1** (a, b), a significant ESA signal is observed between 600 and 800 nm. In THF, this signal corresponds to the PLICT state, which gradually increases in the fs-TAS spectrum and becomes more pronounced in the ns-TA spectrum. In DMSO, the ESA signal is considerably stronger, indicating enhanced stabilization by the more polar solvent. Kinetic analysis (Tables S7 and S8) revealed that the second increase associated with the system’s rotational dynamics occurred much more slowly in DMSO. For example, at 715 and 675 nm, the rise times are 2290 and 357 ps, respectively. By contrast, in THF, the rise time at 650 nm is only 110 ps. These findings support the conclusion that the planarization process in linker **1** is significantly hindered by the higher viscosity of DMSO. It is important to note that due to substantial noise at the fringes of the spectrum, no reliable signal could be extracted below 450 nm. For linker **2**, the appearance is similar to that of the THF spectra except for the appearance of a negative SE signal at 560 nm. The intensity of the SE in DMSO is likely due to the stronger stabilization effect of the more polar solvent.

In the ns-TA spectra, DADS becomes less convoluted as the dynamics approach equilibrium. In Figure S13c, for the ns-TA DADS of linker **1**, an ESA peak decays early at 440 nm within 5.58 ns, and a broad feature is observed from 720 to 840 nm. At 1.12 μs, the intense 420 nm peak decays, along with the broad ESA peak at 645 nm, which we associate with the twisting state for linker **1**. For linker **2**, as shown in Figure S13d, the two intense ESA peaks at 440 and 540 nm decay within 6.76 ns. After 12.2 ns, the two peaks decay almost to equilibrium, revealing a broad SE signal from 480 to 670 nm.

**Figure 5:**
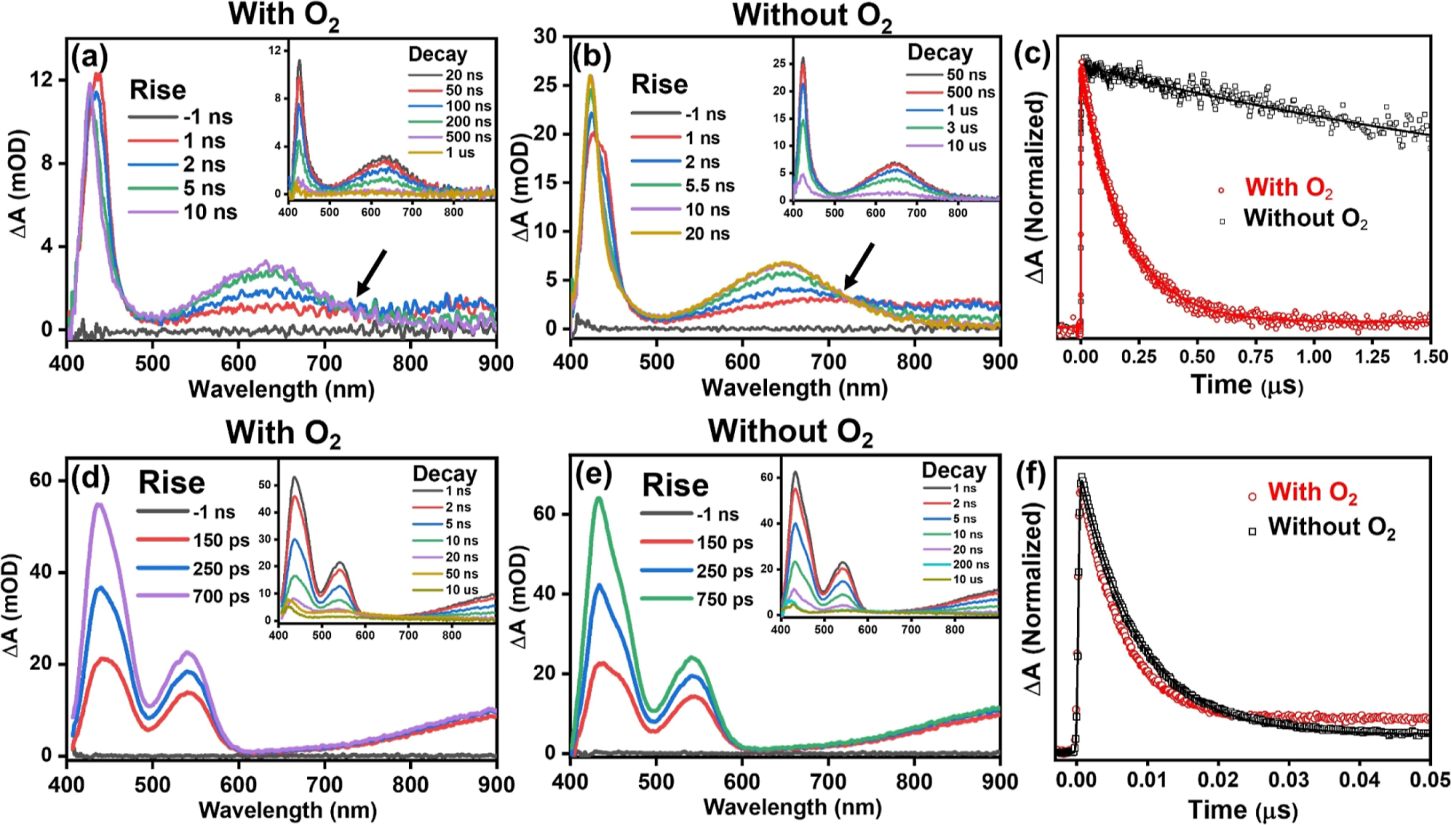
ns-TA spectra at different delay times and associated kinetics for linkers **1** and **2**. (a,b) Spectra for the rise (decay inset) with and without dissolved oxygen for linker **1** (d,e) and linker **2**. (c,f) Kinetic traces at 420 nm with and without oxygen for **1** and **2**, respectively (λ_exc_ = 350 nm).

The possible presence of triplet states for these amine-based linkers can also be deduced from previous reports in the literature on aminoterephthalic acid derivatives in MOFs, which have shown long-lived states in time-resolved spectra. For example, time-resolved vibrational spectroscopy has been used to reveal long-lived transient vibrational states that only relax to the ground state after ∼5 ns for aminoterepthlalic linkers in a TiO_2_ framework.^39^ Low-lying triplet states have also been observed for several aniline derivatives.^40−42^ To comprehensively track the evolution of the TA spectra and investigate the presence of accessible triplet states in Linkers **1** and **2**, ns-TAS was employed due to its ability to monitor long-lasting transient excited states extending into the microsecond range. Figure 5 displays the ns-TA spectra and corresponding kinetics for Linkers **1** and **2**. Both linkers displayed prolonged excited-state absorption signals indicative of potentially accessible triplet states, although only linker **1** persisted into the microsecond time scale. Conducting measurements with and without dissolved oxygen is a well-established method for assessing the existence of a triplet state because it can be quenched by oxygen, thereby increasing the rate of nonradiative deactivation pathways back to the ground state.^43^

In Linker **1**, the spectra revealed three prominent features (Figure 5a,b). These are a sharp excited-state absorption peak at 420 nm, a broad peak centered at 650 nm, and a shorter-lived, very broad signal at 850 nm. Initially, within the first nanosecond, the broad peak at approximately 850 nm began to decay, whereas the 650 nm peak increased, resulting in an isosbestic point at 720 nm. This suggests the formation of a new conformer for linker **1**. The peaks at 420 and 650 nm appear to be due to the peaks that continued beyond the time window of the fs-TA experiment, whereas the broad peak at 850 nm diminished after 2 ns. Additionally, a shoulder peak at 438 nm observed in the fs-TA spectrum disappeared within the 4 ns experimental time frame, which was overshadowed by the stronger 420 nm peak. The two primary features persisted both in the presence and absence of dissolved oxygen and were fitted with a triexponential decay (see Table S1). Notably, the lifetimes of the τ_2_ component showed significant differences, with a shift from 1.5 ± 0.2 μs to 181 ns for the 420 nm peak and from 4.21 ± 0.4 μs to 179 ns for the 650 nm peak, indicating the possible involvement of a triplet state.

For linker **2** (Figure 5d,e), the ns-TA spectra showed a main peak at 432 nm with a shoulder at 455 nm, a less intense peak at 540 nm, and a broad peak extending from 620 to 900 nm. Notably, each peak corresponded to the same peak position as that observed in the fs-TA experiment. The kinetics data for each peak can be found in Tables S4–S6. When the potential for triplet quenching with and without dissolved oxygen was examined, no notable variations in the kinetics were observed. In the absence of O_2_, each peak decayed with a similar biexponential decay, with the major component (τ_1_) of each term ranging between 8.14 and 8.40 ± 0.1 ns. Upon dissolving O_2_ and conducting the measurement again, the kinetics for each peak were described by a triexponential function. The primary component of the fitting for all the peaks decreased consistently within a range of 5.6–6.5 ± 0.08 ns. The additional component (τ_2_) in each fitting could be accurately represented by a feature lasting 115–162 ± 5 ns. The presence of a species displaying a prominent signal on the nanosecond time scale is consistent with the characteristics of a triplet state. Nevertheless, the lack of sensitivity to oxygen quenching implies a more complicated excited-state potential energy landscape.

### 2.4 Excited-State Rotation Dynamics

Taken together, steady-state spectroscopy, excited-state lifetime, and theoretical investigations provide a clearer explanation of the mechanisms underlying the photophysical differences between the two linkers. Beginning with linker **1**, upon excitation, CT occurred from the amine fragment to the whole molecule, as shown for the HOMO–LUMO transition in Figure 1a. As it relaxed from the Franck–Condon state, the molecule became more planar as the two rings twisted toward the optimal dihedral angle of 11°. In the steady-state PL spectrum, a singular broad peak in addition to large Stokes and bathochromic shifts in a strongly polar solvent indicated that the sole emitting state was the CT state.

Initially, the well-studied TICT state was assumed to be the main emissive state. However, in this case, the molecule became increasingly planar in the excited state.^22,38,44,45^ This is in contrast to typical TICT compounds, which are planar in their ground state and become twisted in the excited state. However, certain species can achieve dipole enhancement in the excited state by transitioning from a twisted geometry in the ground state to a more planar geometry.^46^ The transition is often facilitated by enhanced orbital conjugation between separated systems within a single molecule connected by a single rotating bond. This state is known as the planar intramolecular CT state (PLICT).^47^ A singular CT state is consistent with the steady-state PL data in Figure 1c, namely, the large solvochromic redshift in a more polar solvent and increased broadening. Dispersing charge within a molecule increases the variety of orientations that the solvent molecules can adopt to stabilize the resulting charge. This leads to an increase in the inhomogeneous broadening of the PL emission. The lack of secondary peaks or shoulders in the PL spectra, along with the presence of multiple excited-state absorbance (ESA) peaks in the TA spectra, suggests the existence of a range of “dark” or nonemitting excited states.

**Scheme 1:**
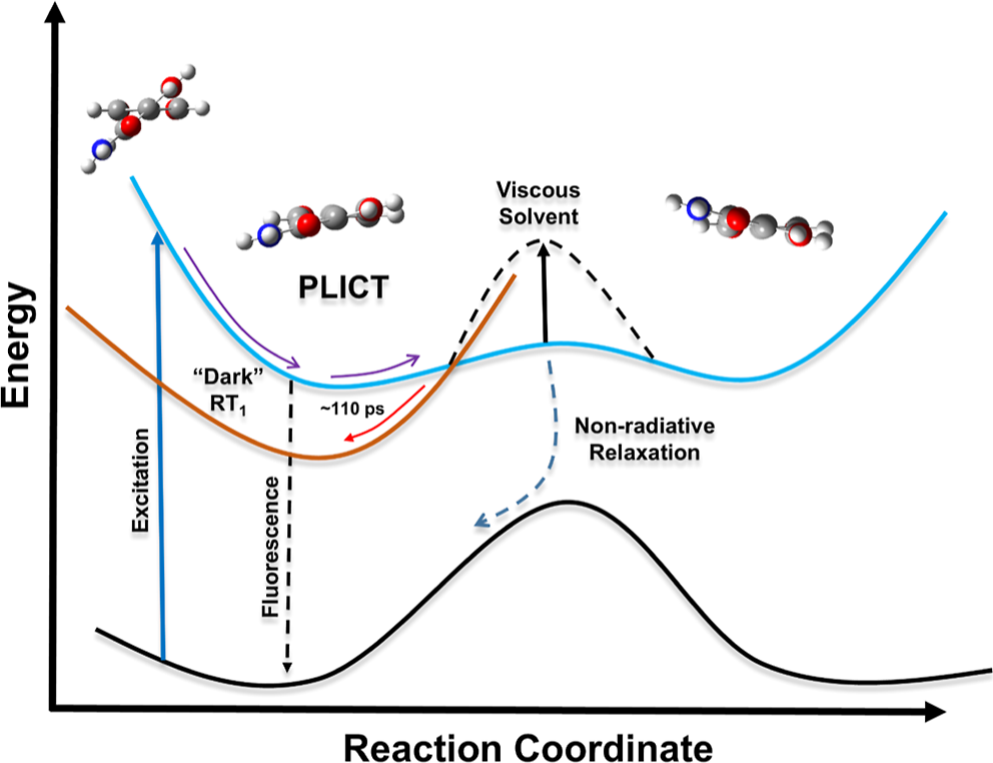
Proposed Approximate Excited-State Dynamics for Linker **1**

The main emitting CT state lasted only approximately 1.6 ns according to the PL lifetime (Figure 2d). However, the two remaining ESA peaks at 418 and 650 nm in ns-TA (Figure 5a) extended into the microsecond time scale. These are likely to be RS_1_ states that decayed via radiationless emission. The long lifetimes and sensitivity of these compounds to dissolved oxygen also suggest that they are likely to be triplet states. The presence of triplet states requires an acceptable orbital transition to overcome the forbidden intersystem crossing. As linker **1** twisted along its shallow well in its PLICT state, it could allow for a spin–orbit manifold for the transition into relaxed triplet states (RT_1_). This is the mechanism behind the uncommon kinetic behavior observed after 110 ps, when the peaks at 418 and 650 nm experienced a simultaneous increase in their intensities. Furthermore, the formation of an entirely new peak at 650 nm suggests a major yet reversible conformational change for **1**. Such an event could only be caused by a change in the excited-state geometry. The large rotational freedom (Δ ≈ 43°) along the central axis indicates that twisting was responsible for access to this new state. The increases in the kinetic intensity could therefore be explained as a redistribution of energy into these RS_1_ states as the conjugation increases between the two ring fragments. The RT_1_ state evolved first; however, a complex manifold must exist between this state and the newly evolved 650 nm state because when the populations of these state start to increase, they do so almost simultaneously. The onset of twisting occurred within the first 110–160 ps according to the kinetics of the two peaks and was in line with the expected time constants reported in the literature.^48,49^ Based on the evidence provided thus far, this is most likely attributed to the constrained rotation caused by the higher viscosity of DMSO compared to THF. This restriction led to a reduction in the radiationless emission in the excited state. The surplus energy was preserved in a PLICT state, enhancing the photoluminescent pathway. A diagram illustrating the evolution of the excited state for linker **1** is depicted in Scheme 1. The absence of a scheme for linker **2** is due to the uncertainty of the early triplet dynamics.lightFor linker **2**, a similar CT event occurred that involved charge transfer from the amine-containing fragment to the LUMO, which was spread across the whole molecule (Figure 1b). The increased intensity and width of the peak were absent in this case, with the bathochromic shift occurring when the sample was changed to a more polar solvent being the only notable difference. linker **2** is relatively rigid because of steric hindrance. Therefore, unsurprisingly there was no change in the PL peak intensity or width. Linker **2** became slightly more planar in the excited state, attaining the optimal dihedral angle of 40.9°. However, it was restricted to the variation of the twist angle by ∼25° at RTP. Therefore, it was unlikely that the CT was associated with a TICT- or PLICT-type phenomenon. In previous studies, the stabilization of TICT states was often attributed to the presence of specific structural features, such as a substantial twist or rotational freedom between the donor and acceptor moieties.^45,50^ This twist can stabilize the excited state through reduced conjugation or altered electronic interactions, making the TICT state more energetically favorable. For linkers **1** and **2**, the absence of significant TICT state stabilization can be attributed to their molecular structure. These linkers may lack the necessary conformational flexibility or electronic environment that favors the twisted geometry. The increased planarization observed in our DFT calculations suggests that excited-state stabilization is achieved through a different mechanism, possibly involving stronger conjugation or less electronic decoupling compared to the conventional systems that exhibit TICT behavior.

The lack of any secondary peaks or shoulders in the PL spectrum also indicated the presence of dark nonemitting excited states, as the TA spectrum (Figure 4b) contained several peaks. The fs- and ns-TA results revealed similar spectral features extending to the nanosecond time scale, indicating that there was no significant change in geometry, as shown in linker **1**. Factoring in the TCSPC lifetime for linker **2**, there was a slight increase in the lifetime from 3.29 to 3.91 ns (Figure 2b,d) when the solution was changed to DMSO, indicating a slight reduction in nonradiative emission, as variation in a small range of twist angles was still allowed in the excited state. These results indicate that the high PL for linker **2** originates from the restricted access to the radiationless decay channels caused by the steric hindrance of the amino group at the meta position.

The insignificant difference between the lifetimes for linker **2** with and without dissolved oxygen despite long lifetimes extending into the nanosecond time scale was unexpected. However, the molecule likely relaxed from a triplet state before significant quenching could occur. Fitting of the kinetics revealed a triexponential decay for the dissolved oxygen sample compared with a biexponential decay without dissolved oxygen. The new time component present for each peak in the ns-TA spectrum ranged from 115 to 162 ns and was within the order of the τ_2_ time constant for linker **1** when quenched with O_2_ (179–181 ns). Therefore, quenching likely occurred when a majority of the excited-state population had already returned to the ground state. Notably, the quenching rate may also have been influenced by the viscosity and dielectric constant of the solvent.

## 3 Conclusions

In this study, we explored the impact of intramolecular ring rotation on the PL and lifetime of two organic free linkers. The comprehensive analysis of the steady state, excited-state lifetime, and theoretical investigations provided a deeper understanding of the complex photophysical differences observed for the two distinct linkers.

For linker **1**, excitation triggered a CT from the amine fragment to the entire molecule, resulting in a distinct PLICT state. While the steady-state PL indicated a singular CT state, time-resolved measurements revealed the presence of long-lived dark LE states with triplet character. Complex kinetics and conformational alterations suggest a diverse array of states, with twisting motions playing a significant role in accessing these states. The changes in the PL intensity due to solvent dependence were attributed to the enhancement of the typical photoluminescent pathway by restricted rotation and increased viscosity.

For linker **2**, a similar CT occurred with minimal impact on the PL peak intensity or width, underscoring the structural rigidity imposed by steric hindrance. The absence of secondary peaks in the PL spectrum indicated the presence of dark nonemitting excited states, which was supported by consistent spectral features in the time-resolved measurements. The steric hindrance, which limited access to nonradiative decay pathways, contributed to the increased PL observed for linker **2**. The minor difference between the lifetimes with and without dissolved oxygen indicated that relaxation from the triplet state occurred before quenching.

In summary, the study of the spectroscopic characteristics and dynamic behaviors of Linkers **1** and **2** provided a nuanced understanding of their photophysical properties. The analysis of steady-state absorption and emission spectra, along with TD-DFT calculations, elucidated the CT nature of the transitions and underscored the considerable influence of solvent polarity and amino group position on molecular electronic structures.

## 4 Experimental

### 4.1 Steady-State Measurements

Steady-state absorption spectroscopy was performed using a Cary-5000 UV–Vis spectrometer (Varian). PL measurements were performed using a Fluoromax-4 fluorimeter (Horiba) with an excitation wavelength of 350 nm for both linker **1** (OD: 0.39) and linker **2** (OD: 0.50). Liquid samples were prepared in a 1 cm quartz cuvette.

### 4.2 Time-correlated Single-Photon Counting

The time-resolved PL data for emission decays were measured using the TCSPC technique in a Halcyone setup (Ultrafast Systems). The samples were excited with a 350 nm pulsed laser beam from an optical parametric amplifier (Newport Spectra Physics) seeded with an Astrella femtosecond pulsed laser (150 fs, 800 nm, 1 kHz, Coherent). The emissions from the samples were collected and collimated by several parabolic mirrors. Then, they were passed through 400 nm long-pass filters to eliminate the remaining light from excitation. The detection wavelength for the emission of both linkers was 460 nm in DMSO and 440 nm in THF. The excitation power was controlled with a set of variable neutral density filters to ensure that fewer than 1% of the excitation events resulted in a detected photon. The PL signal was then focused on an optical fiber and directed to a monochromator and a PMT detector. The resolution of the equipment was better than 150 ps.

### 4.3 Computational Methods

Electronic structure calculations were performed using the DFT and TD-DFT methods. The ground-state geometries for linkers **1** and **2** were optimized using the CAM-B3LYP functional together with the 6-311++G(d,p) Popple basis set. The THF solvent was modeled using the IEFPCM approximation.^51^ Frequency calculations for each geometry confirmed the minima. The excited-state optimized geometries were also computed at the same level of theory and confirmed by frequency calculations. Both systems were calculated with the same DFT and TD-DFT levels of theory. The values of the carbon–carbon bond orders based on Laplacian bond order analysis were computed using the Multiwfn package for the ground- and excited-state optimized structures.^35^

### 4.4 fs Transient Absorption

Fs-TA experiments were performed using a Helios spectrometer (Ultrafast Systems). The beam from an Astrella Ti:Sapph (Coherent) pulsed laser (150 fs, 1 kHz, 800 nm, 7 mJ/pulse) was split and directed to an optical parametric amplifier (Newport Spectra Physics) to tune the excitation wavelength to 350 nm. This beam was focused on the sample after it was passed through a mechanical chopper (500 Hz). The other 800 nm branch of the beam proceeded to a delay stage to obtain the temporal resolution and was then focused on a CaF_2_ crystal to generate a white-light probe. The white light was split into a reference channel and overlapped in the sample with the pump beam. The absorption change was measured with respect to the time delay and wavelength.

### 4.5 ns Transient Absorption

For the ns-TA measurements, an EOS setup (Ultrafast Systems) was used, with a 350 nm pump pulse generated after passing through an 800 nm beam (150 fs pulse, 1 kHz, 7 mJ/pulse, Astrella; Coherent) into the spectrally tunable optical parametric amplifier focused on the samples. A subnanosecond pulsed white-light probe beam from a commercial source (Ultrafast Systems) was split into two parts: the first was used as a reference, and the second was directed and focused on the sample, which was spatially overlapped with the pump beam and then directed to a second detector. The system monitored the fluctuations in the probe beam intensity, the pump–probe delay was controlled electronically, and the maximum time window was close to half the repetition period of the pump laser from a ns to a microsecond time scale. The results were fitted using the algorithm included in the Ultrafast Systems Software. The detailed experimental setup of the EOS can be found elsewhere.^52^ To remove oxygen from the sample, fresh samples were placed in a glovebox under a nitrogen atmosphere and dissolved in degassed THF. To dissolve the oxygen, air was bubbled into the cuvette using a 10 mL syringe twice directly after the first measurement without oxygen.

### 4.6 Materials

All reagents were used as received from commercial sources without further purification. The linkers were purchased from Jilin Chinese Academy of Science–Yanshen Technology Co., Ltd. and were used without further purification.

## Supporting Information Available

It contains electron density differences from GS to ES, experimental and calculated UV–vis spectra, fs/ns-TA spectra for linkers **1** and **2**, time constants for the fittings, DAD spectra from the global analysis and concentration-dependent TCSPC lifetime measurements (PDF)
