# Supplementary material for: Excited-State Rotational Dynamics of Amine-Functionalized Terephthalic Acid Derivatives as Linker Models for Metal–Organic Frameworks
Source: J Phys Chem A. 2025 Jan 15;129(4):836–47. doi: 10.1021/acs.jpca.4c03827 (PMC11789137; doi:10.1021/acs.jpca.4c03827)
Supplement: Supplementary file 1 — jp4c03827_si_001.pdf [file jp4c03827_si_001.pdf]

Supporting Information for

Excited-State Rotational Dynamics of Amine-functionalized Terephthalic Acid Derivatives as Linker Models for Metal–Organic Frameworks

George Healing [1,2], Maksim Zakharzhevskii [2], Issatay Nadinov [1], Luis Gutiérrez-Arzaluz [1,2], Shorooq A. Alomar [1], Jorge Gascon [2] and Omar F. Mohammed* [1,2]

[1]Advanced Membranes and Porous Materials Center, Division of Physical Science and Engineering, King Abdullah University of Science and Technology, Thuwal 23955-6900, Kingdom of Saudi Arabia

[2]KAUST Catalysis Center, Division of Physical Sciences and Engineering, King Abdullah University of Science and Technology, Thuwal 23955-6900, Kingdom of Saudi Arabia

The Förster-Hoffmann[1] equation provides an empirical relationship between fluorescence lifetime (τ) and viscosity (η):

$$\tau = C\eta^n$$

where C is a proportionality constant, and n is the power-law exponent that reflects the sensitivity of the molecule's intramolecular motions to the solvent viscosity. The log plot:

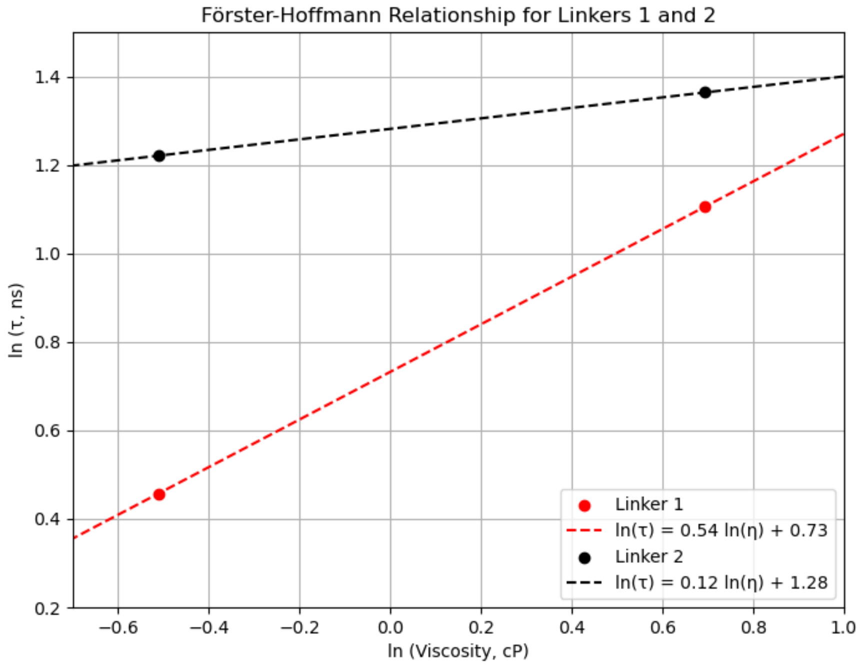

Figure S1. Log plot of fluorescence lifetime (ln(τ)) versus solvent viscosity (ln(η)) for Linker **1** and Linker **2**, demonstrating the application of the Förster-Hoffmann equation. Linear fits reveal power-law exponents of n=0.54 (Linker **1**) and n=0.12 (Linker **2**), reflecting differences in their sensitivity to viscosity

For Linker **1** (measured in THF and DMSO, viscosities 0.6 cP and 2.0 cP, respectively), the power-law exponent n = 0.54 was determined, indicating a moderate dependence of the fluorescence lifetime on solvent viscosity. Linker **2** in the same solvents, yields n = 0.12, suggesting a weaker dependence of fluorescence lifetime on viscosity for this molecule.

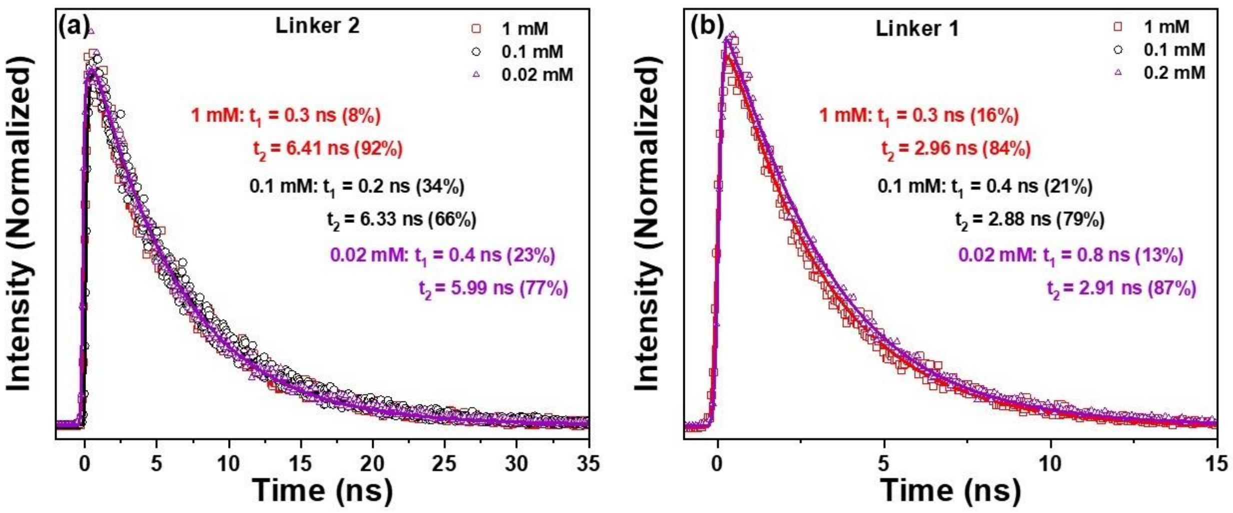

Figure S2. Normalized PL lifetime measurements for Linker **2** (a) and Linker **1** (b) with their respective biexponential time constants fitting values at concentrations varying from 0.02 mM to 1mM. (λ_{exc} = 350 nm)

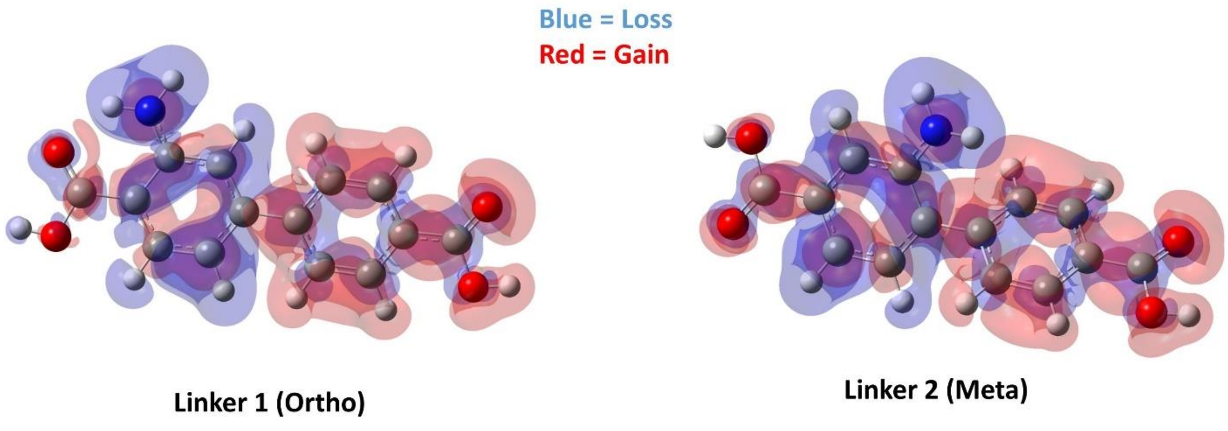

Figure S3. Optimized structures showing electron density changes (blue = loss/red = gain) upon the vertical electronic transition to the first singlet excited state.

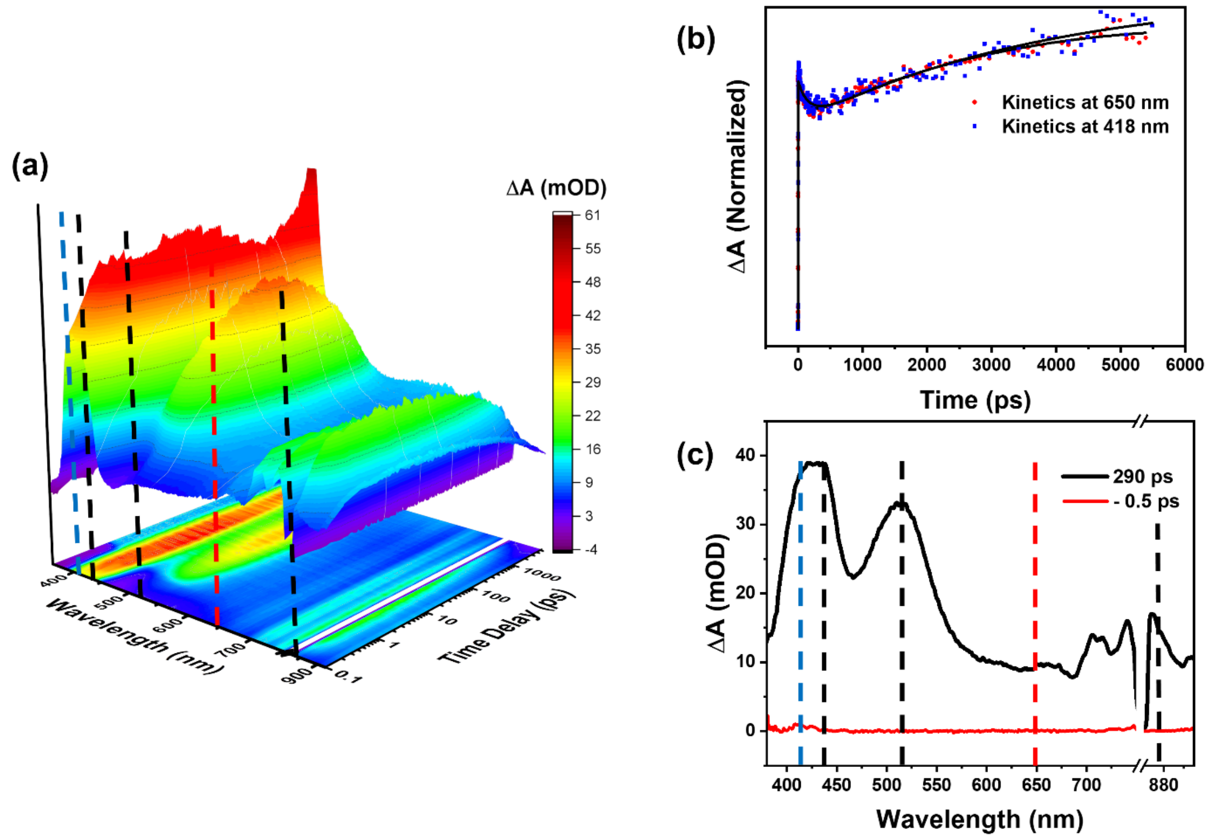

Figure S4. (a) 3D color contour plot of the fs-TA spectra for Linker **1**, (b) A comparison of the rise feature after 110 ps and (c) a standard TA spectrum at 290 ps is shown for clarity. (λ_{exc} = 350 nm)

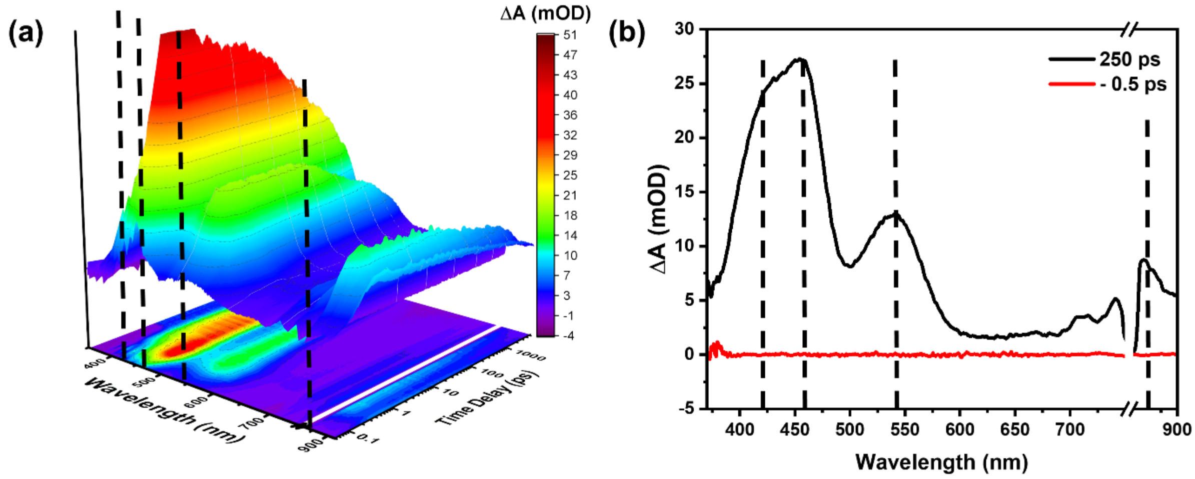

Figure S5. (a) 3D color contour plot of the fs-TA spectra for Linker **2** with (b) a standard TA spectrum at 250 ps is shown for clarity. (λ_{exc} = 350 nm)

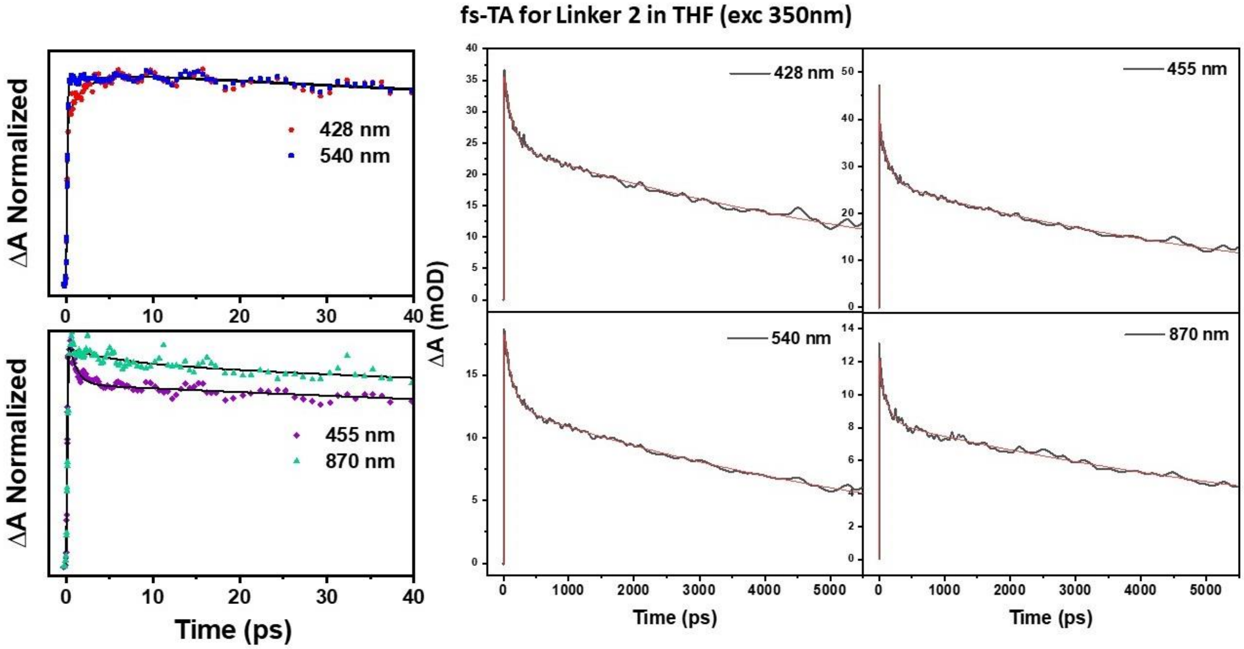

Figure S6. Fs-TA kinetics traces for the 4 representative peaks of Linker **2** (right), and the early times details normalized on the left. (λ_{exc} = 350 nm)

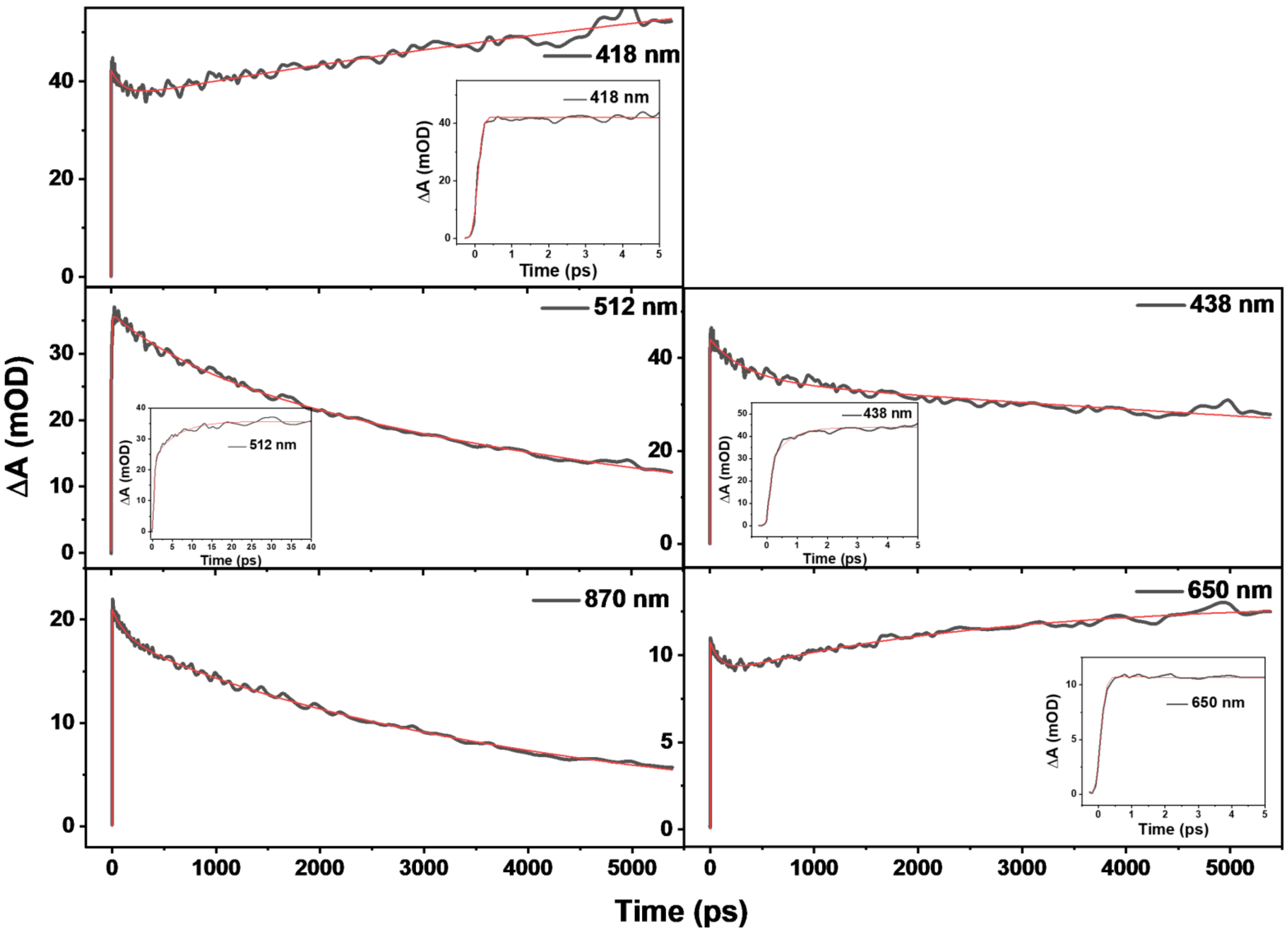

Figure S7. Fs-TA kinetics profiles for the 5 representative peaks of Linker **1**, and the early times details in the insets. (λ_{exc} = 350 nm)

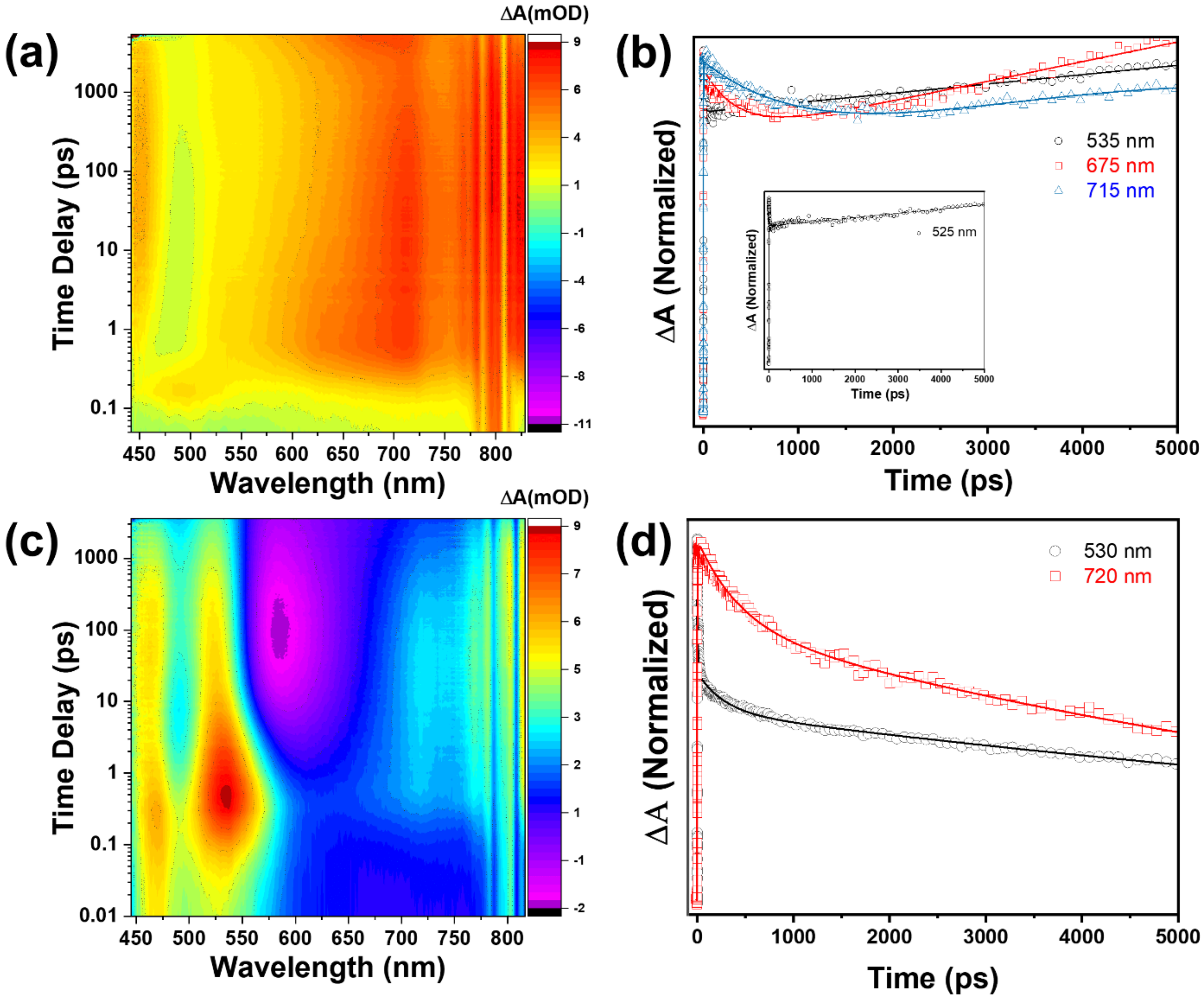

Figure S8. 2D color contour plots of the fs-TA spectra for the linkers in DMSO. Linker **1** (a) with (b) a kinetic plot comparing peaks at 535, 675 and 715 nm (535 nm is highlighted inset) and (c) Linker **2** with (d) a kinetic plot comparing peaks at 530 and 720 nm. (λ_{exc} = 350 nm)

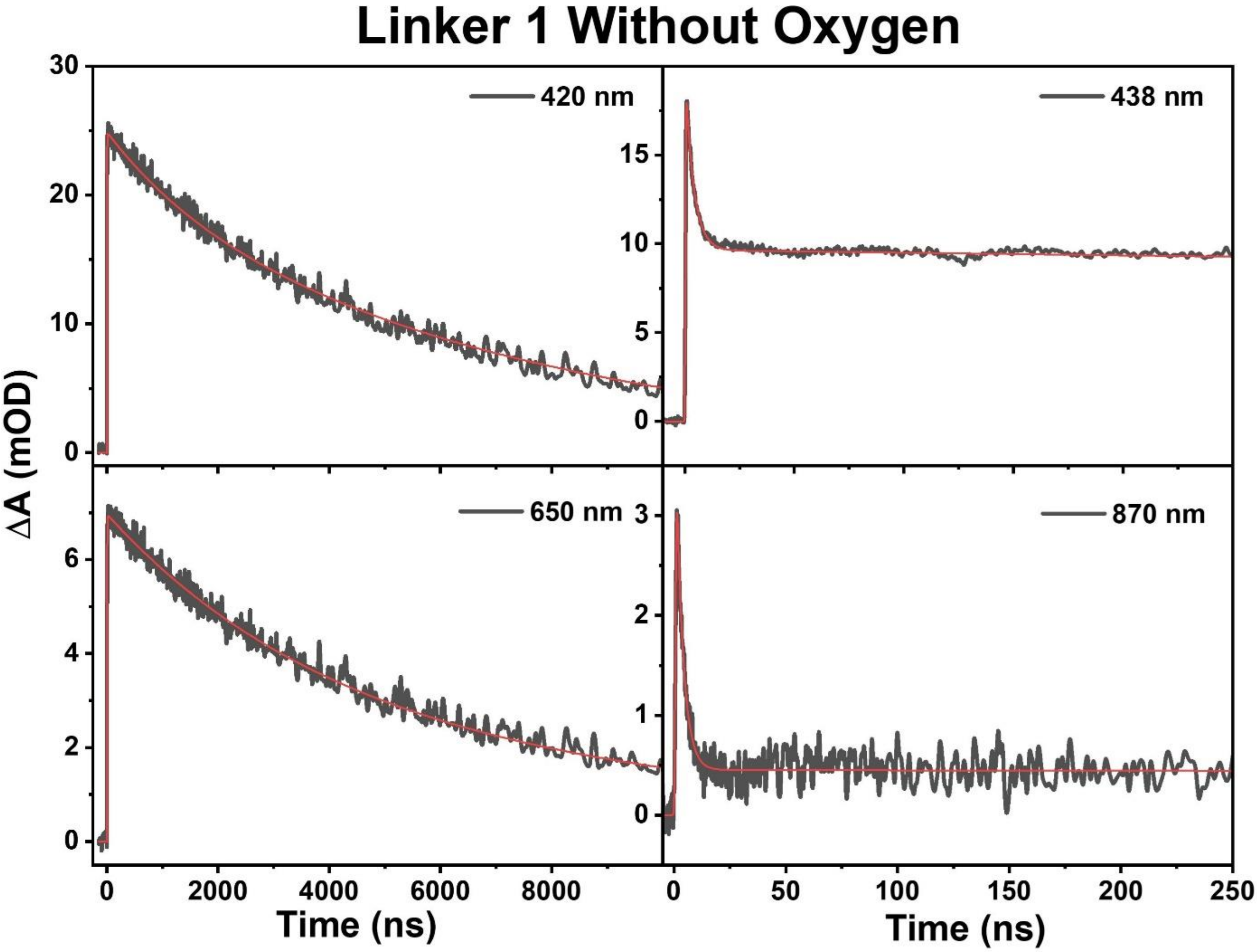

Figure S9. ns-TA kinetics traces for the 4 peaks of Linker **1** in solutions without oxygen. (λ_{exc} = 350nm)

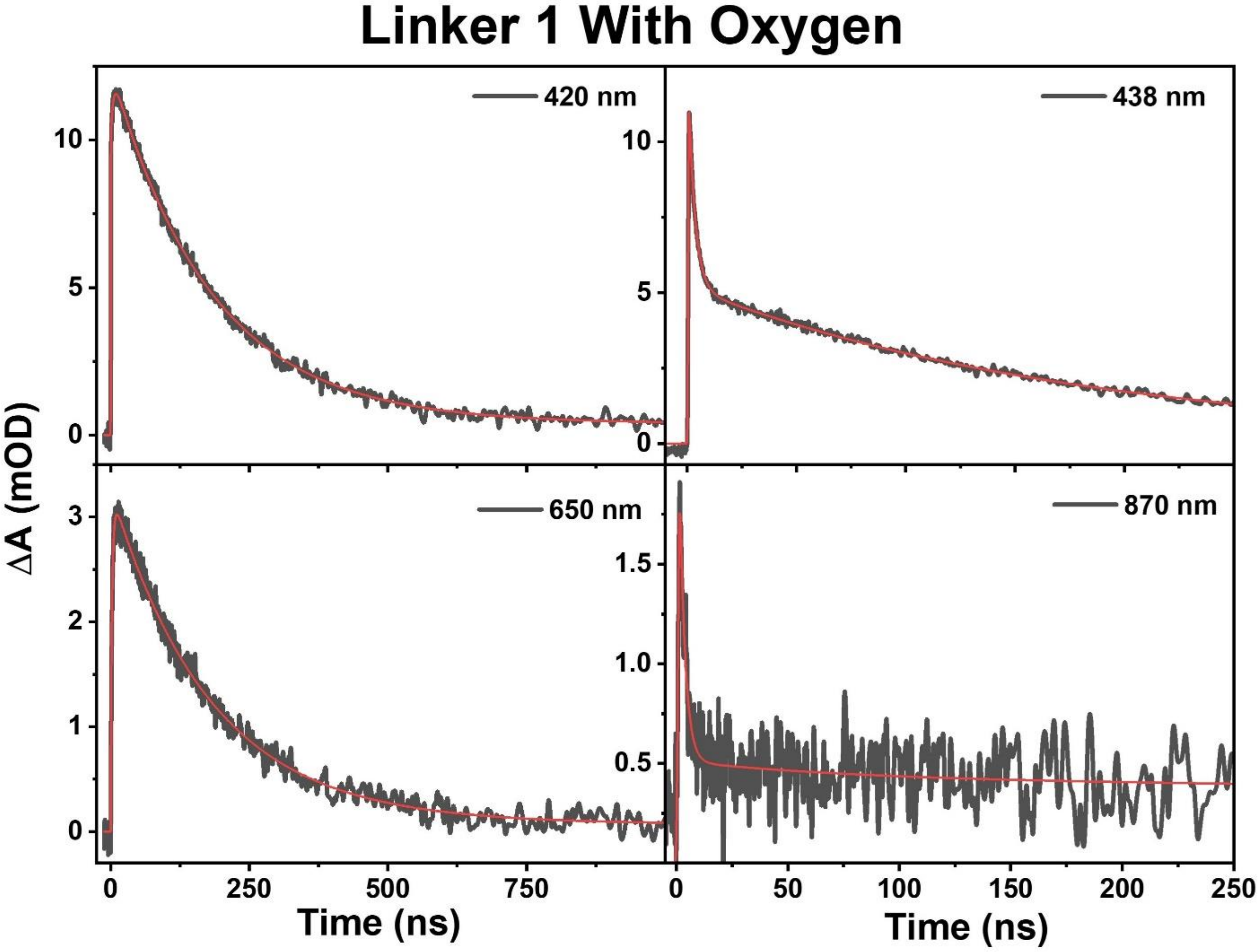

Figure S10. ns-TA kinetics traces for the 4 peaks of Linker **1** in solutions with oxygen. (λ_{exc} = 350nm)

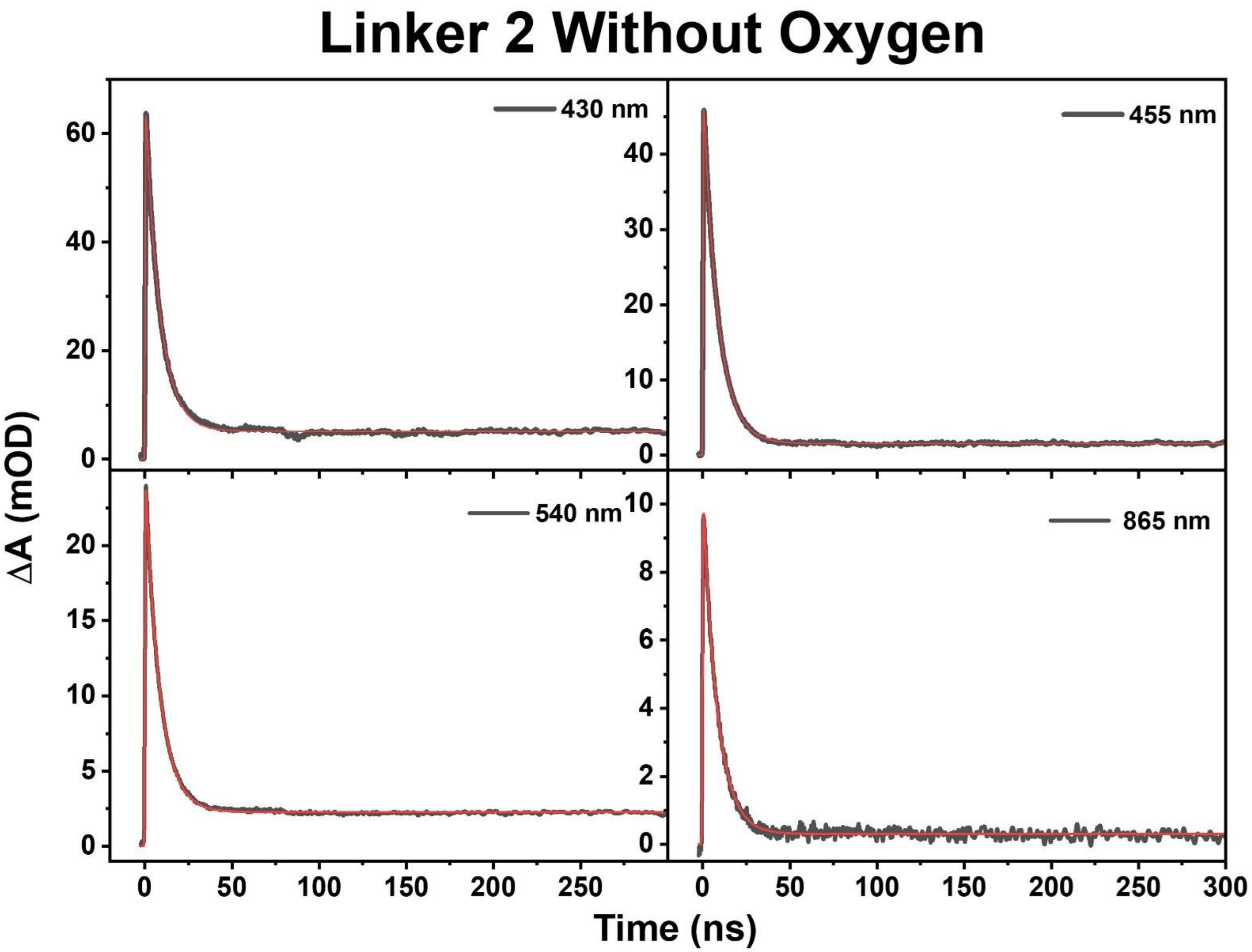

Figure S11. ns-TA kinetics traces for the 4 peaks of Linker **2** in solutions without oxygen. (λ_{exc} = 350nm)

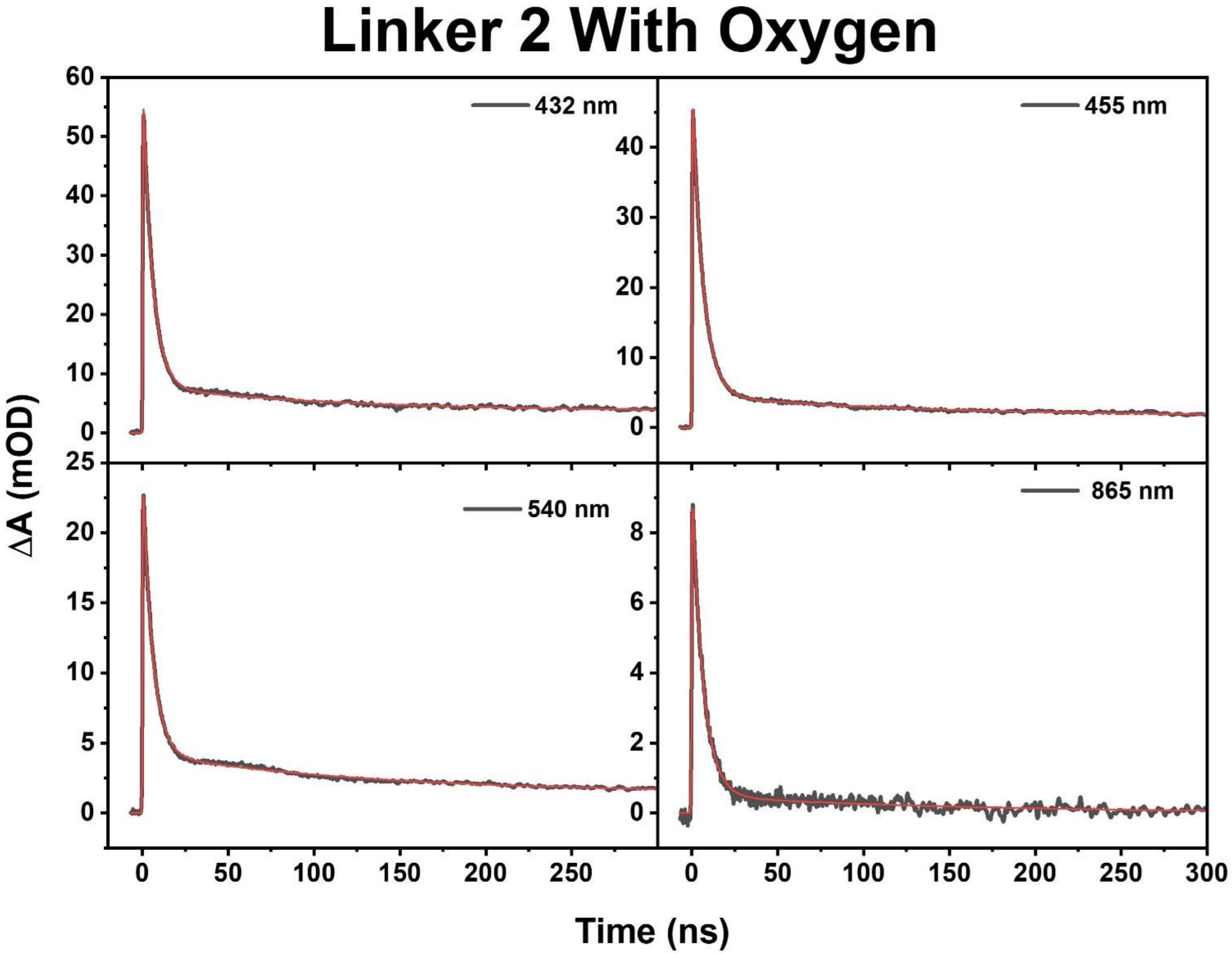

Figure S12. ns-TA kinetics traces for the 4 peaks of Linker **2** in solutions with oxygen. (λ_{exc} = 350nm)

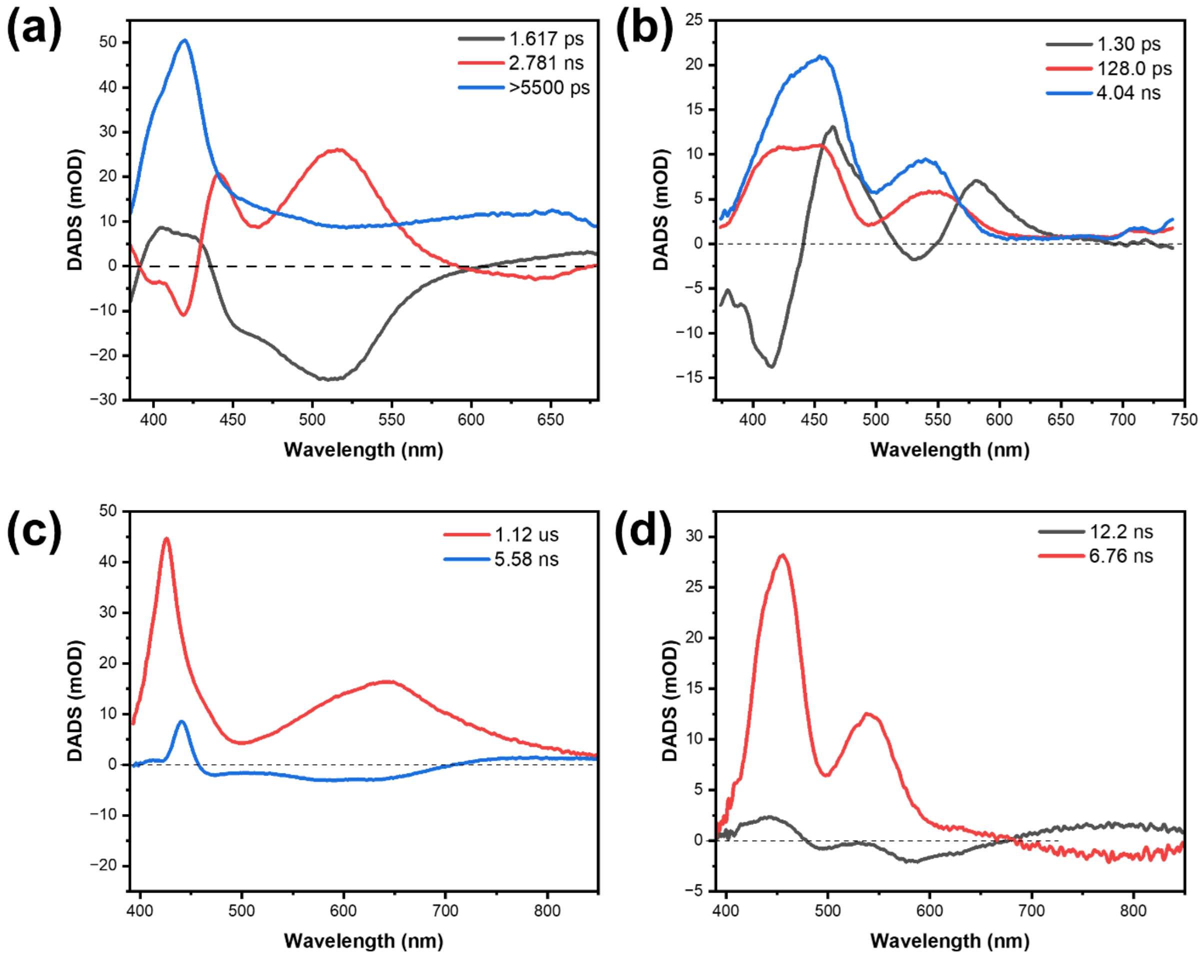

Figure S13. Global Analysis (DADS) of the fs-TA spectra for the Linker **1** (a) and Linker **2** (b). The same analysis for the ns-TA spectra for Linker **1** (c) and Linker **2** (d).

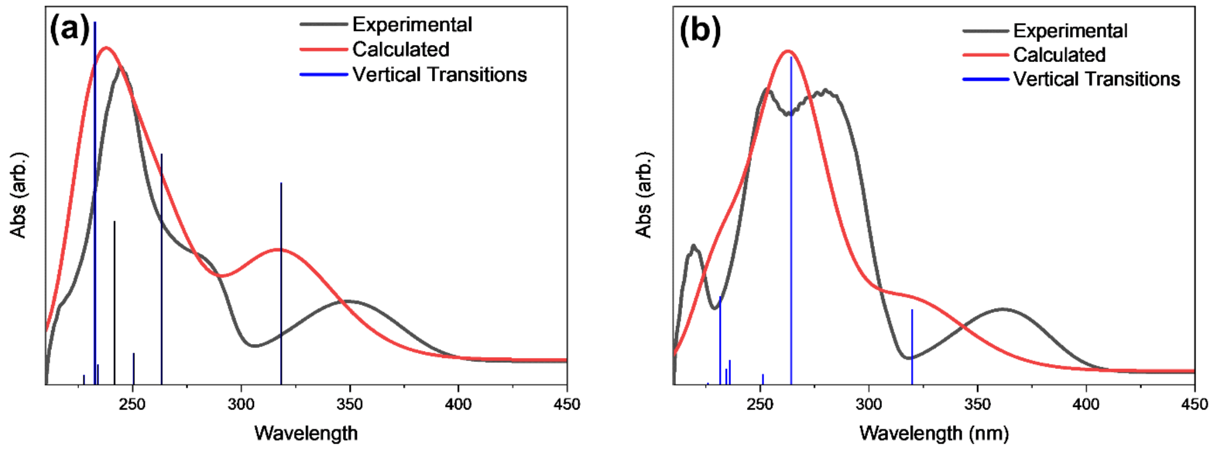

Figure S14. Comparison between experimental and calculated UV-Vis spectra for Linker 1 (a) and Linker 2 (b).

Table S1. Time constants for the fs-TA exponential fittings for Linker **1** in THF.

Wavelength	a_1	τ_1 (ps)	a_2	τ_2 (ps)	a_3	τ_3 (ps)
418 nm	0.07	160 ± 64	-0.27	4760 ± 340	0.66	> 5500
438 nm	-0.19	1.08 ± 0.3	0.15	273 ± 63	0.66	17700 ± 2480
512 nm	-0.25	16.5 ± 4.3	0.12	112 ± 54	0.62	4940 ± 223
650 nm	0.1	109 ± 23	-0.22	4430 ± 630	0.68	> 5500
870 nm	-0.08	0.8 ± 0.6	0.13	145 ± 35	0.78	4390 ± 156

Table S2. Time constants for the ns-TA exponential fittings for Linker **1** without oxygen in THF.

Wavelength	a_1	τ_1 (ns)	a_2	τ_2 (µs)	a_3	τ_3 (µs)
420 nm	-0.2	3.6 ± 0.4	0.13	1.5 ± 0.2	0.64	7 ± 0.3
438 nm	0.5	3.8 ± 0.2	0.5	6.35 ± 0.08		
512 nm						
650 nm	-0.39	3.5 ± 0.3	0.5	4.21 ± 0.4	0.16	19.2 ± 7
870 nm	0.89	3.1 ± 0.4	0.12	13.6 ± 2.8		

Table S3. Time constants for the ns-TA exponential fittings for Linker **1** with oxygen in THF.

Wavelength	a_1	τ_1 (ns)	a_2	τ_2 (ns)	a_3	τ_3 (ns)
420 nm	-0.17	4.2 ± 0.3	0.81	181 ± 42	0.02	20
438 nm						
512 nm						
650 nm	-0.4	2.8 ± 0.4	0.57	179 ± 53	0.02	4.3
870 nm						

Table S4. Time constants for the fs-TA exponential fittings for Linker **2** in THF.

Wavelength	a_1	τ_1 (ps)	a_2	τ_2 (ps)	a_3	τ_3 (ps)
430 nm	-0.182	1.9 ± 0.5	0.26	139 ± 16	0.55	>5500
455 nm	0.22	0.932 ± 0.186	0.24	139 ± 14	0.54	>5500
540 nm	-0.05	5.02 ± 3.1	0.31	137 ± 12	0.63	>5500
865 nm	0.06	6.04 ± 3.1	0.26	125 ± 25	0.68	>5500

Table S5. Time constants for the ns-TA exponential fittings for Linker **2** without oxygen in THF.

Wavelength	a_1	τ_1 (ns)	a_2	τ_2
432 nm	0.92	8.17 ± 0.1	0.08	>5500
455 nm	0.97	8.40 ± 0.02	0.03	>5500
540 nm	0.9	8.17 ± 0.05	0.1	>5500
865 nm	0.97	8.14 ± 0.1	0.03	>5500

Table S6. Time constants for the ns-TA exponential fittings for Linker **2** with oxygen in THF.

Wavelength	a_1	τ_1 (ns)	a_2	τ_2 (ns)	a_3	τ_3
432 nm	0.87	5.6 ± 0.07	0.07	115 ± 7	0.06	>5500
455 nm	0.91	6.2 ± 0.04	0.06	129 ± 6	0.03	>5500
540 nm	0.83	5.7 ± 0.06	0.1	132 ± 4	0.07	>5500
865 nm	0.95	6.5 ± 0.02	0.04	162 ± 4	0.01	>5500

Table S7. Time constants for the fs-TA exponential fittings for Linker **1** in DMSO.

Wavelength	a_1	τ_1 (ps)	a_2	τ_2 (ps)	a_3	τ_3 (ns)
535 nm	0.55	0.178 ± 0.06	0.18	10.1 ± 1.1	0.27	>4
675 nm	0.51	0.178 ± 0.04	0.17	357	0.32	>4
715 nm	0.58	0.178 ± 0.05	0.11	2290	0.31	>4

Table S8. Time constants for the fs-TA exponential fittings for Linker **2** in DMSO.

Wavelength	a_1	τ_1 (ps)	a_2	τ_2 (ps)	a_3	τ_3 (ns)	a_4	τ_4 (ns)
530 nm	0.55	0.178 ± 0.08	0.18	5.1 ± 0.77	0.5	274	0.23	>4
720 nm	0.48	0.178 ± 0.11	0.17	7.0 ± 1.01	0.11	445	0.23	>4
